# Peripheral ERK modulates acupuncture-induced brain neural activity and its functional connectivity

**DOI:** 10.1038/s41598-021-84273-y

**Published:** 2021-03-04

**Authors:** Ji-Yeun Park, Seong-Jin Cho, Soon-Ho Lee, Yeonhee Ryu, Jae-Hwan Jang, Seung-Nam Kim, Hi-Joon Park

**Affiliations:** 1 College of Korean Medicine, Daejeon University, 62 Daehak-ro, Dong-gu, Daejeon, 34520 Republic of Korea; 2 Clinical Medicine Division, Korea Institute of Oriental Medicine, 1672 Yuseong-daero, Yuseong-gu, Daejeon, 34054 Republic of Korea; 3 Acupuncture and Meridian Science Research Center, Kyung Hee University, 26 Kyungheedae-ro, Dongdaemoon-gu, Seoul, 02447 Republic of Korea; 4 College of Korean Medicine, Dongguk University, 32 Dongguk-Ro, Goyang, 10326 Republic of Korea

**Keywords:** Computational biology and bioinformatics, Neuroscience, Physiology, Medical research

## Abstract

Acupuncture has been widely used as a therapeutic intervention, and the brain network plays a crucial role in its neural mechanism. This study aimed to investigate the acupuncture mechanism from peripheral to central by identifying how the peripheral molecular signals induced by acupuncture affect the brain neural responses and its functional connectivity. We confirmed that peripheral ERK activation by acupuncture plays a role in initiating acupuncture-induced peripheral proteomic changes in mice. The brain neural activities in the neocortex, hippocampus, thalamus, hypothalamus, periaqueductal grey, and nucleus of the solitary tract (Sol) were significantly changed after acupuncture, and these were altered by peripheral MEK/MAPK inhibition. The arcuate nucleus and lateral hypothalamus were the most affected by acupuncture and peripheral MEK/MAPK inhibition. The hypothalamic area was the most contributing brain region in contrast task PLS analysis. Acupuncture provoked extensive changes in brain functional connectivity, and the posterior hypothalamus showed the highest betweenness centrality after acupuncture. After brain hub identification, the Sol and cingulate cortex were selected as hub regions that reflect both degree and betweenness centrality after acupuncture. These results suggest that acupuncture activates brain functional connectivity and that peripheral ERK induced by acupuncture plays a role in initiating brain neural activation and its functional connectivity.

## Introduction

Acupuncture works by inserting and/or manipulating a needle at a specific point called the acupuncture point (acupoint). It produces therapeutic effects in various diseases, including pain-related disease^1,2^ and neurodegenerative disease^3–5^. Although the clinical effectiveness of acupuncture has been well identified^2,5^, and its fundamental mechanisms are being actively investigated^6,7^, the underlying mechanism of acupuncture action remains unclear.

In determining the mechanism of acupuncture treatment, it is essential to distinguish between the specific and nonspecific effects, also known as the placebo effect of acupuncture. Since the placebo effect is mainly due to psychological mechanisms, it is necessary to study the mechanisms that result from the physical stimuli applied to specific acupoints to distinguish the specific effect of acupuncture^8^. Several brain imaging studies have shown that specific changes in brain neural activation reflect the specific effect of acupuncture^9–11^. Recently, it has been known that brain neuronal connectivity, a useful method to clarify the functional network of the brain, plays a crucial role in explaining the neural mechanism of acupuncture^12,13^. It has been used in imaging-based approaches^14^ and electroencephalography analysis to detect coordinated activity across distributed and spatially remote brain regions^15,16^. As the demand for more direct analysis of the neural activity in the brain, c-Fos, an immediate early gene, has been used as a surrogate marker for neuronal activity. Increased neuronal activity will produce an up-regulation of c-Fos^17,18^, and subsequent computation of interregional correlations can be analysed to identify a network that is active after acupuncture.

The clinical effect of acupuncture is manifested through several stages of biologic changes. These changes are first initiated in the peripheral skin tissues and/or muscles around the acupoints that were stimulated with acupuncture needles. Several studies have speculated that local responses such as TRPV1^19,20^ or adenosine A1 receptor^21^ play a crucial role in triggering the analgesic effects of acupuncture. In our previous study, we identified peripheral ERK signaling, a major molecule activated on the skin tissue after an acupuncture treatment, had a crucial role in mediating the analgesic effect of acupuncture^22^. However, the mechanisms for how the peripheral signals expressed by acupuncture are connected to the central neural changes are still not elucidated. Thus, it is necessary to investigate the whole-brain response after acupuncture and the link between the central nervous and peripheral molecular signals.

Therefore, the purpose of this study was to investigate the acupuncture mechanism from peripheral to central by identifying how the peripheral ERK signal induced by acupuncture affects the neural responses and functional connectivity in the brain after acupuncture.

## Results

### Peripheral ERK inhibition blocked the acupuncture-increased thermal pain threshold in normal mice

**Figure 1:**
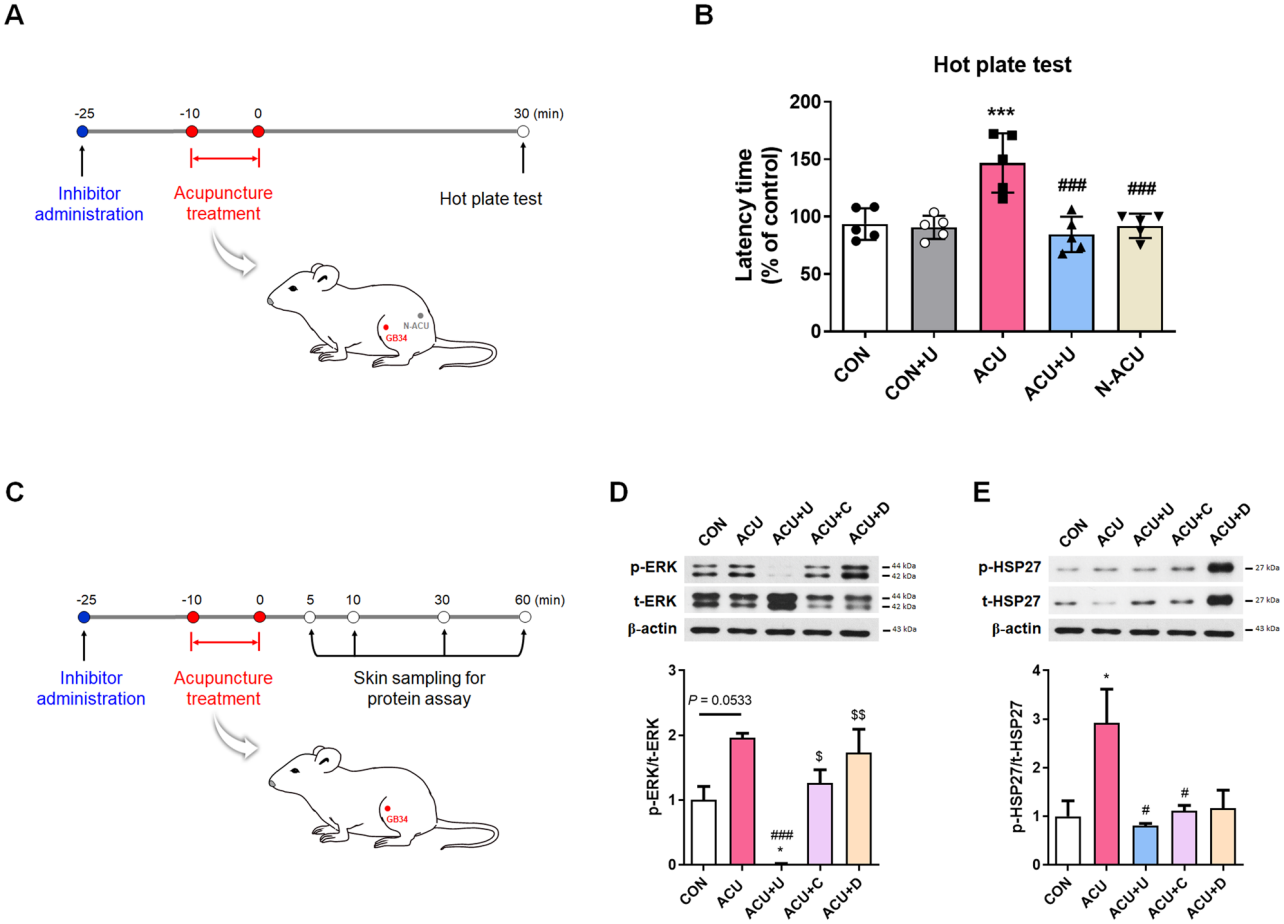
The role of peripheral ERK in the acupuncture-induced thermal pain threshold changes and the acupuncture-induced peripheral molecular signals. (**A**,**B**) Peripheral administration of U0126, an ERK inhibitor, before acupuncture treatment significantly blocked the effect of acupuncture in increasing the thermal pain threshold (each n = 5). (**C**,**D**) The activated p-ERK after acupuncture treatment was inhibited by U0126; however, it was not affected by CPZ, a TRPV1 inhibitor and DPCPX, an A1R inhibitor. The activated p-HSP27 were inhibited by U0126, CPZ, and DPCPX (each n = 3). The western blot image represent the cropped blots. Full length blots are presented in Supplementary Figure S2. *CON* control, *CON + U* U0126 administration, *ACU* acupuncture treatment, *ACU* *+* *U* U0126 administration followed by acupuncture treatment, *ACU + C* CPZ administration followed by acupuncture treatment, *ACU + D* DPCPX administration followed by acupuncture treatment, *N-ACU* acupuncture treatment at non-acupoint on the hips. *P < 0.05, ***P < 0.001 compared to the CON group, ^#^*P* < 0.05, ^###^*P* < 0.001 compared to the ACU group, ^$^*P* < 0.05, ^$$^*P* < 0.01 compared to the ACU + U group. One-way ANOVA was followed by the Newman–Keuls post-hoc test. Data are expressed as the mean ± SEM.

Our previous study^22^ identified that mechanical hyperalgesia altered by acupuncture in a mouse pain model was blocked by peripheral U0126 administration, an ERK inhibitor. In this study, we investigated whether the effect of acupuncture on increasing pain threshold in normal mice is also regulated by peripheral ERK blockade. We administered U0126 to acupoints 15 min before acupuncture treatment to block ERK expression by acupuncture. Then, we treated acupuncture at the GB34 acupoint, and the thermal pain threshold was evaluated using the hot plate test 30 min later. We found a significant increase in latency time in the acupuncture treated group (ACU) compared to the control group (CON; F_4, 20_ = 12.42, *P* < 0.001) and non-acupoint treated group (N-ACU; F_4, 20_ = 12.42, *P* < 0.001). Peripheral administration of U0126 before acupuncture treatment significantly blocked the effect of acupuncture in increasing the thermal pain threshold (F_4, 20_ = 12.42, *P* < 0.001 in ACU vs. ACU + U) (Fig. 1A,B). During the experiments, no adverse events were observed. From these results, we could reconfirm that acupuncture-induced peripheral ERK activation plays an important role in mediating the therapeutic effect of acupuncture.

### Identifying the importance of ERK signaling in the acupuncture mechanism

Previously, we found that peripheral ERK was activated prominently in the early part of stimuli-transmission after acupuncture treatment^22^. Although it can be assumed that the initially expressed ERK plays an important role in initiating the acupuncture effect, the role of other molecules expressed by acupuncture should also be considered. Accordingly, we screened the expression of other molecules after the acupuncture treatment, and in particular, we observed the expression of molecules related to ERK activation. We performed two-dimensional electrophoresis (2-DE) to derive acupuncture-induced protein candidates in skin tissue. About 500 spots were changed 5 or 30 min after the acupuncture treatment, and 11 spots were remarkably detected. Protein identification of the 11 spots was performed by peptide mass fingerprinting (PMF). Keratin, apolipoprotein A-1, beta-actin, serum albumin, heat shock protein (HSP), transferrin, transketolase, and peroxiredoxin-1 (PRDX1) were top-scored or highly scored protein candidates (Supplementary Table 1). Of these proteins, we focused on HSP and PRDX1, a protein associated with not only ERK signaling but also cell signaling transduction. The degree of protein expression was measured at each time point after acupuncture treatment. We found that the activation of phospho-ERK (p-ERK) was increased significantly 5 min after acupuncture treatment compared to the CON group (F_4,10_ = 20.04, *P* < 0.05), and it decreased from 10 min later (Supplementary Fig. 1A). The activation of phospho-HSP27 (p-HSP27) was also increased 5 min after acupuncture treatment compared to the CON group (F_4,10_ = 3.107, *P* = 0.1394), and it decreased from 10 min later (Supplementary Fig. 1B). In contrast, the activation of PRDX1 decreased 5 min after acupuncture treatment than that in the CON group (F_4,10_ = 0.8736, *P* = 0.5513), and it was restored gradually (Supplementary Fig. 1C). Based on these results, we focused on the activation of p-ERK and p-HSP27 after acupuncture as an essential part of initiating the acupuncture effect.

Then, we analyzed the correlation between ERK expression and other molecules found in this study or known to be expressed after acupuncture. Since the activation of A1R and TRPV1 after acupuncture treatment was found to be necessary for producing acupuncture analgesia^19–21^, we investigated the effects of ERK, TRPV1, and A1R inhibitors (U0126, CPZ, and DPCPX) on acupuncture induced p-ERK, p-HSP27 in the skin layer (Fig. 1C–E). The activated p-ERK after acupuncture treatment (F_4, 10_ = 13.20, *P* = 0.0533 vs. CON) was inhibited by peripheral U0126 administration (F_4, 10_ = 13.20, *P* < 0.001) and it was not affected by CPZ or DPCPX (Fig. 1D). The activated p-HSP27 after acupuncture treatment (F_4, 10_ = 5.027, *P* < 0.05 vs. CON) were inhibited by peripheral U0126 administration (F_4, 10_ = 5.027, *P* < 0.05), CPZ (F_4, 10_ = 5.027, *P* < 0.05), and DPCPX (F_4, 10_ = 5.027, *P* = 0.0545) (Fig. 1E). These results indicate that the p-ERK activation following acupuncture is the most essential part in initiating the effect of acupuncture.

### Brain neural activation after acupuncture treatment was regulated by peripheral ERK signaling

**Figure 2:**
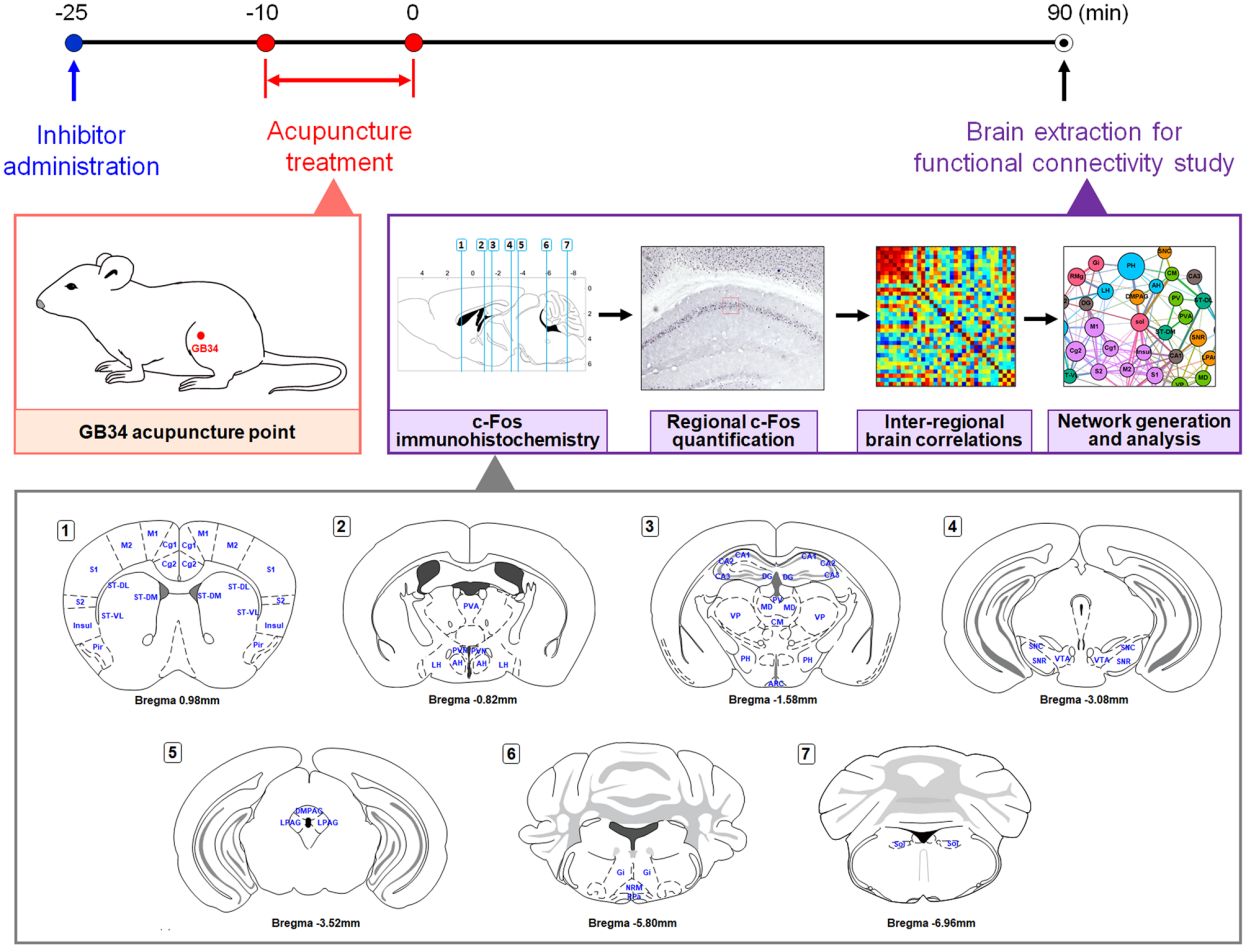
Schematic diagram of experimental schedule for functional connectivity study. Acupuncture was performed at bilateral GB34 (Yangneungcheon, Yanglingquan) for 10 min and inhibitor was administrated 15 min before acupuncture treatment. The brains were removed 90 min after acupuncture treatment for identifying the brain neural responses and the brain functional connectivity. The c-Fos positive cells were quantified in each of the 34 brain regions, and a set of inter-regional correlations were computed. Finally, functional networks were generated. Abbreviations for each brain region are shown in Table 1.

To investigate the scientific mechanism of acupuncture from peripheral to central, we first identified the changes in brain neural activity after acupuncture treatment. Then, we identified the correlation between acupuncture-activated peripheral ERK signaling and brain neural activity. Acupuncture was performed on the GB34 acupoints, and the c-Fos positive cells were activated in thirty-four brain regions of the cortex, cerebral nuclei, thalamus, hypothalamus, hippocampus, midbrain, and medulla were analysed. The details of acupuncture treatment and the location of 34 brain regions are shown in Fig. 2. After acupuncture treatment, the number of c-Fos-positive cells was significantly increased in the primary somatosensory cortex (S1; F_2, 20_ = 9.008, *P* < 0.01), secondary motor cortex (M2; F_2, 21_ = 18.98, *P* < 0.001), cingulate cortex area 2 (Cg2; F_2, 21_ = 11.34, *P* < 0.001), insular cortex (Insul; F_2, 20_ = 7.958, *P* < 0.01), piriform cortex (Pir; F_2, 21_ = 7.598, *P* < 0.01), lateral hypothalamus (LH; F_2, 22_ = 21.53, *P* < 0.001), arcuate nucleus (ARC; F_2, 23_ = 26.32, *P* < 0.001), dorsomedial periaqueductal grey (DMPAG; F_2, 22_ = 6.995, *P* < 0.05), lateral periaqueductal grey (LPAG; F_2, 24_ = 19.751, *P* < 0.001), and nucleus of solitary tract (Sol; F_2, 17_ = 6.783, *P* < 0.01) compared to the CON group. These signals were all diminished after peripheral U0126 administration (F_2, 20_ = 9.008, *P* < 0.05 in S1; F_2, 21_ = 18.98, *P* < 0.01 in M2; F_2, 21_ = 11.34, *P* < 0.01 in Cg2; F_2, 20_ = 7.958, *P* < 0.05 in Insul; F_2, 21_ = 7.598, *P* < 0.05 in Pir; F_2, 22_ = 21.53, *P* < 0.001 in LH; F_2, 23_ = 26.32, *P* < 0.001 in ARC; F_2, 22_ = 6.995, *P* < 0.01 in DMPAG; F_2, 24_ = 19.75, *P* < 0.001 in LPAG; F_2, 17_ = 6.783, *P* < 0.05 in Sol) than in the ACU group.

**Figure 3:**
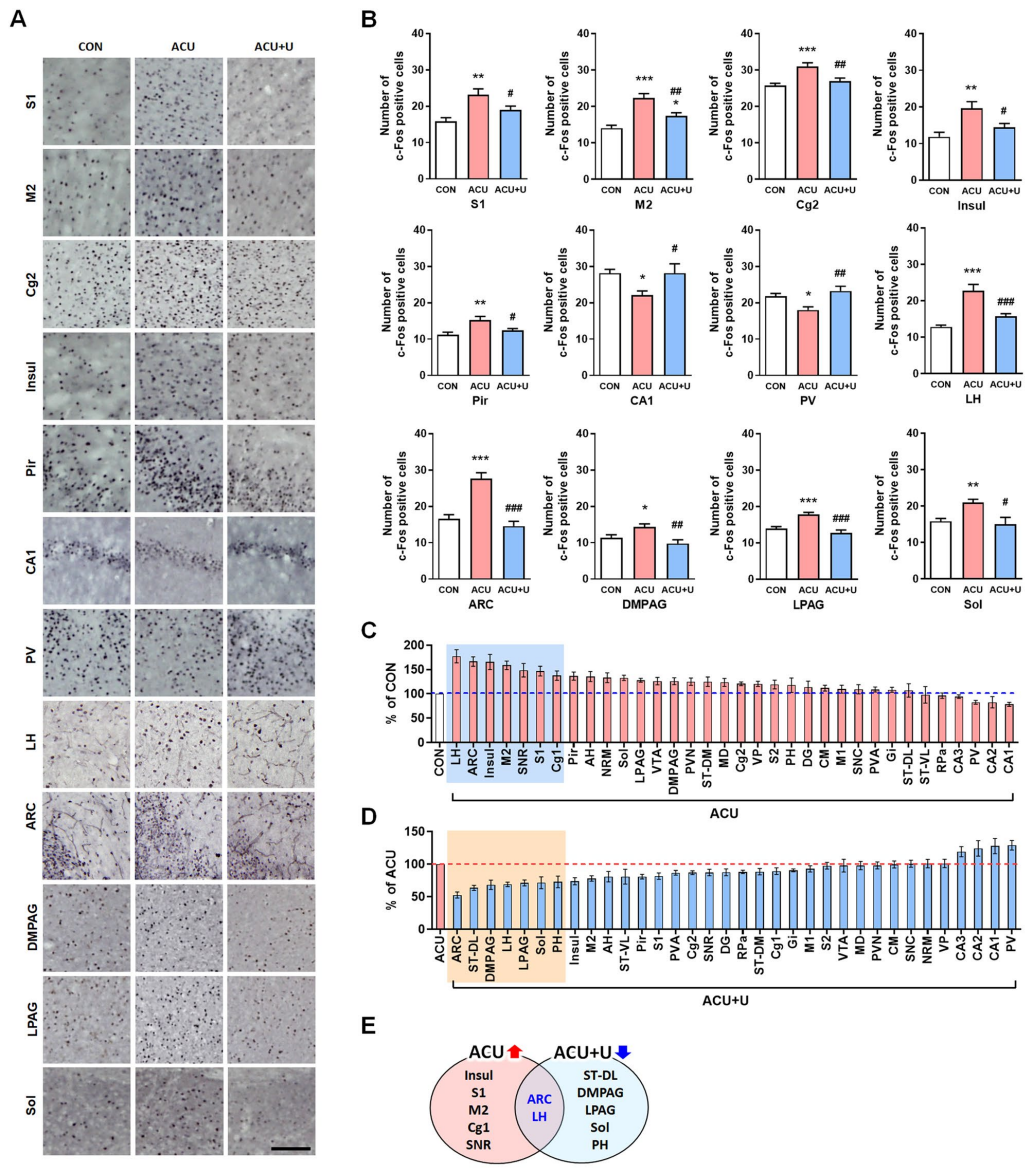
Expression of c-Fos after acupuncture treatment expressed in each brain region. (**A**,**B**) The number of c-Fos-positive cells changed after acupuncture or acupuncture with U0126 administration. (**C**) Increased percentage of c-Fos-positive cells after acupuncture treatment compared to the control group. (**D**) Decreased percentage of c-Fos-positive cells after U0126 administration followed by acupuncture treatment compared to the acupuncture treatment only. (**E**) The overlap between brain regions ranked above the 80th percentile for acupuncture increased, and U0126 decreased-c-Fos activation. *CON* control, *ACU* acupuncture treatment, *ACU* + *U* U0126 administration followed by acupuncture treatment. Abbreviations for each brain region are shown in Table 1. *P < 0.05, **P < 0.01, ***P < 0.001 compared to the CON group. ^#^*P* < 0.05, ^##^*P* < 0.01, ^###^*P* < 0.001 compared to the ACU group. One-way ANOVA was followed by the Newman–Keuls post-hoc test. Data are expressed as the mean ± SEM. The number of animals in each group is shown in Table 1.

After acupuncture treatment, the number of c-Fos-positive cells was significantly decreased in the CA1 of the hippocampus (CA1; F_2, 22_ = 5.004, *P* < 0.05) and paraventricular thalamic nucleus posterior (PV; F_2, 25_ = 7.549, *P* < 0.05) compared to the CON group, and these changes were inhibited after peripheral U0126 administration (F_2, 22_ = 5.004, *P* < 0.05 in CA1; F_2, 25_ = 7.549, *P* < 0.01 in PV) compared to the ACU group (Fig. 3A,B).

In some brain regions, increased c-Fos activation after acupuncture was not altered by U0126 administration. The number of c-Fos positive cells was significantly increased in the cingulate cortex area 1 (Cg1; F_2, 21_ = 7.019, *P* < 0.01), paraventricular hypothalamic nucleus (PVN; F_2, 18_ = 4.250, *P* < 0.05), raphe magnus nucleus (RMg; F_2, 20_ = 6.762, *P* < 0.01) compared to the CON group. However, these signals was not altered by U0126 administration (F_2, 21_ = 7.019, *P* < 0.05 in Cg1; F_2, 18_ = 4.250, *P* < 0.05 in PVN; F_2, 20_ = 6.762, *P* < 0.05 in RMg when compared between CON vs. ACU + U) (Table 1). The c-Fos expression in the whole brain regions and the results of one-way ANOVA are shown in Table 1.

**Table 1:** Abbreviations of brain regions and the number of c-Fos-positive cells in each brain region.

No	Brain region	Abbreviation	Number of c-Fos positive cells					
			CON (n)		ACU (n)		ACU + U (n)	
**Cortex**								
1	Primary somatosensory cortex	S1	15.8 ± 1.0	(10)	23.2 ± 1.6**	(6)	18.9 ± 1.1^#^	(7)
2	Secondary somatosensory cortex	S2	16.7 ± 1.0	(10)	19.9 ± 1.5	(6)	19.4 ± 1.0	(7)
3	Primary motor cortex	M1	14.7 ± 1.0	(10)	16.1 ± 1.3	(7)	14.9 ± 0.8	(7)
4	Secondary motor cortex	M2	14.1 ± 0.8	(10)	22.3 ± 1.3***	(7)	17.4 ± 0.9*^,##^	(7)
5	Cingulate cortex, area 1	Cg1	16.0 ± 0.9	(10)	21.9 ± 1.6**	(7)	19.6 ± 1.1*	(7)
6	Cingulate cortex, area 2	Cg2	25.7 ± 0.7	(10)	31.0 ± 1.1***	(7)	26.9 ± 0.9^##^	(7)
7	Insular cortex	Insul	11.9 ± 1.2	(10)	19.6 ± 1.8**	(6)	14.4 ± 1.1^#^	(7)
8	Piriform cortex	Pir	11.2 ± 0.7	(10)	15.3 ± 1.0**	(7)	12.4 ± 0.6^#^	(7)
**Cerebral nuclei**								
9	Caudate putamen (striatum)-dorsal medial	ST-DM	19.0 ± 1.0	(10)	23.7 ± 2.0*	(7)	21.0 ± 1.2	(7)
10	Caudate putamen (striatum)-dorsal lateral	ST-DL	7.4 ± 0.7	(10)	7.9 ± 1.1	(7)	5.0 ± 0.3	(7)
11	Caudate putamen (striatum)-ventral lateral	ST-VL	3.8 ± 0.6	(10)	3.7 ± 0.7	(7)	3.0 ± 0.4	(7)
**Hippocampus**								
12	Field CA1 of hippocampus	CA1	28.1 ± 1.0	(9)	22.1 ± 1.1*	(9)	28.1 ± 2.6^#^	(7)
13	Field CA2 of hippocampus	CA2	5.5 ± 0.5	(10)	5.0 ± 0.4	(10)	6.2 ± 0.6	(7)
14	Field CA3 of hippocampus	CA3	20.8 ± 1.1	(10)	19.6 ± 0.7	(10)	23.3 ± 1.5	(7)
15	Dentate gyrus	DG	7.3 ± 0.7	(10)	8.3 ± 0.9	(10)	7.2 ± 0.4	(7)
**Thalamus**								
16	Paraventricular thalamic nucleus anterior	PVA	20.1 ± 1.2	(10)	21.9 ± 1.1	(10)	19.0 ± 0.8	(6)
17	Paraventricular thalamic nucleus posterior	PV	21.8 ± 0.7	(9)	18.0 ± 0.8*	(10)	23.2 ± 1.2^##^	(9)
18	Cetral medial thalamic nucleus	CM	19.6 ± 1.0	(10)	21.9 ± 1.1	(10)	21.8 ± 1.4	(9)
19	Mediodorsal thalamic nucleus	MD	14.9 ± 0.8	(10)	18.4 ± 1.2	(10)	18.0 ± 1.1	(9)
20	Ventral posterior thalamic nucleus	VP	14.7 ± 1.0	(10)	17.7 ± 0.8	(10)	17.8 ± 1.3	(9)
**Hypothalamus**								
21	Paraventricular hypothalamic nucleus	PVN	38.0 ± 2.0	(8)	47.5 ± 2.7*	(8)	46.5 ± 2.3*	(5)
22	Anterior hypothalamic area	AH	10.8 ± 0.6	(9)	14.63 ± 0.9*	(8)	11.8 ± 1.3	(8)
23	Lateral hypothalamic area	LH	12.8 ± 1.5	(9)	22.8 ± 1.5***	(8)	15.7 ± 0.8^###^	(8)
24	Arcuate hypothalamic nucleus	ARC	10.5 ± 1.1	(10)	12.4 ± 1.2***	(8)	9 ± 1.4^###^	(8)
25	Posterior hypothalamic area	PH	16.6 ± 1.4	(10)	27.6 ± 0.3	(8)	14.6 ± 1.2	(8)
**Midbrain**								
26	Dorsomedial periaqueductal gray	DMPAG	11.4 ± 0.7	(9)	14.3 ± 0.7*	(9)	9.8 ± 1.0^##^	(7)
27	Lateral periaqueductal gray	LPAG	14.0 ± 0.5	(10)	17.9 ± 0.6***	(10)	12.7 ± 0.8^###^	(7)
28	Substantia nigra reticular	SNR	5.2 ± 0.6	(10)	7.7 ± 0.7*	(10)	6.7 ± 0.4	(6)
29	Substantia nigra compacta	SNC	8.5 ± 0.4	(10)	9.3 ± 0.8	(10)	9.4 ± 0.5	(7)
30	Ventral tegmental area	VTA	9.4 ± 0.5	(9)	11.9 ± 0.7*	(10)	11.6 ± 1.1	(6)
**Medulla**								
31	Gigantocellular reticular nucleus	Gi	13.7 ± 0.7	(9)	14.8 ± 0.7	(8)	13.4 ± 0.3	(7)
32	Raphe pallidus nucleus	RPa	13.0 ± 0.8	(9)	12.5 ± 0.8	(8)	11.0 ± 0.3	(5)
33	Raphe magnus nucleus	RMg	11.6 ± 0.5	(9)	15.4 ± 1.2**	(8)	15.5 ± 1.0*	(6)
34	Nucleus of solitary tract	Sol	15.8 ± 0.8	(10)	21.0 ± 0.6**	(5)	15.0 ± 1.6^#^	(5)

Thirty-four brain regions for c-Fos quantification. Brain regions are categorized by major brain subdivisions. *P < 0.05, **P < 0.01, ***P < 0.001 compared to the CON group. ^#^*P* < 0.05, ^##^*P* < 0.01, ^###^*P* < 0.001 compared to the ACU group. *CON* control, *ACU* acupuncture treatment, *ACU + U* U0126 administration followed by acupuncture treatment, *n* number. One-way ANOVA was followed by the Newman–Keuls post-hoc test. Data are expressed as the mean ± SEM.

To identify the brain areas that were most affected by acupuncture and peripheral U0126 administration, the c-Fos changes in the ACU and ACU + U groups were sorted in order of change. LH, ARC, Insul, M2, substantia nigra reticular (SNR), S1, and Cg1 were ranked above the 80th percentile in c-Fos increase after acupuncture treatment compared to the control. ARC, dorsal lateral part of striatum (ST-DL), DMPAG, LH, LPAG, Sol, and posterior hypothalamic area (PH) were ranked above the 80th percentile in the c-Fos decrease of the ACU + U group compared to the ACU group (Fig. 3C,D). Then, the overlap between brain regions ranked above the 80th percentile for acupuncture increased, and U0126 decreased- c-Fos activation was selected. ARC and LH in the hypothalamic area were the most affected brain regions by acupuncture and peripheral U0126 administration, followed by acupuncture (Fig. 3E).

### Contrast task PLS analysis after acupuncture

**Figure 4:**
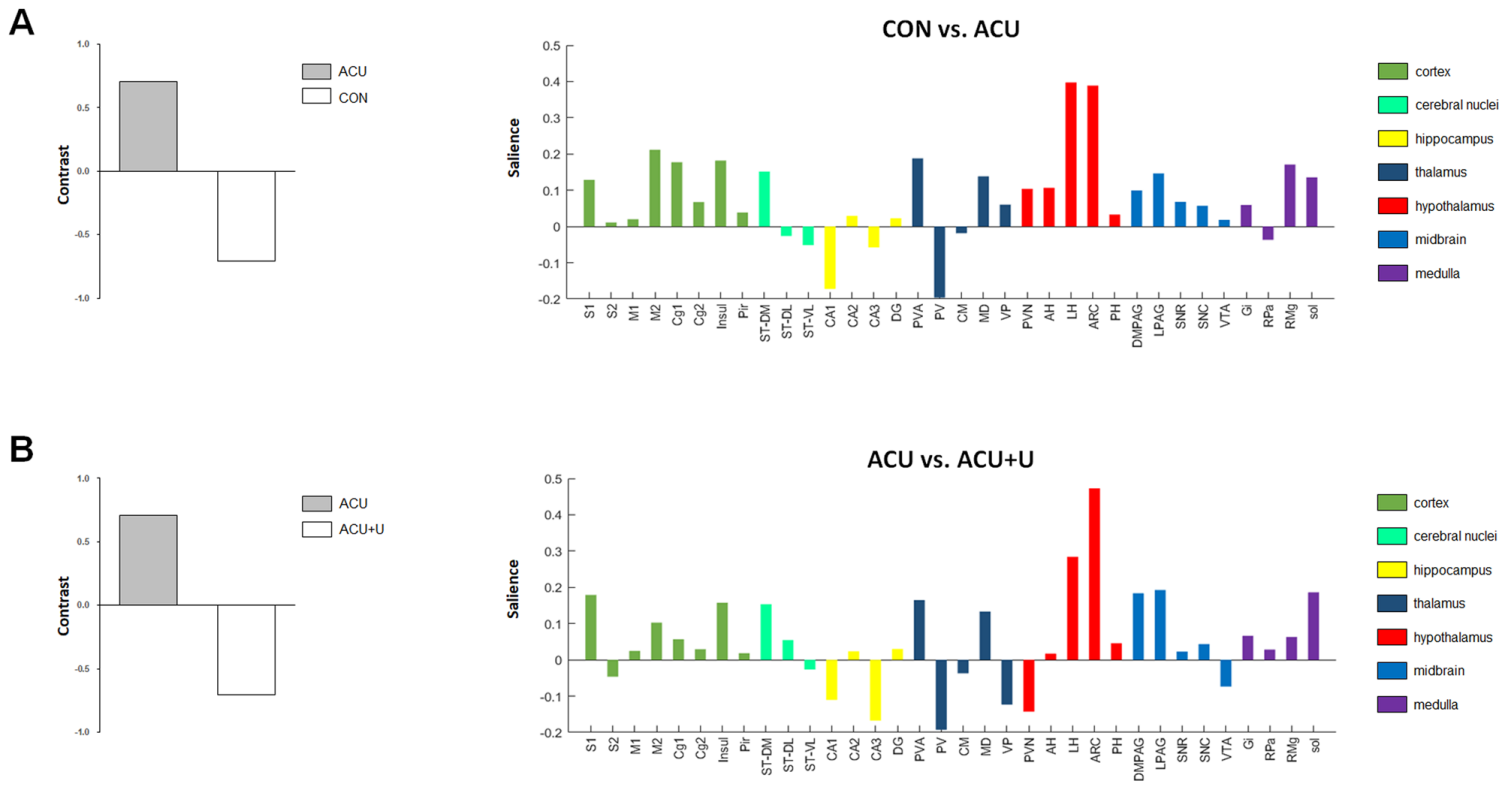
Contrast task PLS analysis of brain c-Fos expression in CON vs. ACU and ACU vs. ACU + U. (**A**,**B**) Two pairs of ACU vs. CON and ACU vs. ACU + U were compared in the Contrast task PLS analysis of c-Fos expression. The contrast (left) reflects the experimental condition in CON vs. ACU (**A**) and ACU vs. ACU + U (**B**). Salience scores (right) identified that the brain regions which differed in c-Fos expression between the experimental conditions were intensely similar between the ACU vs. CON and ACU vs. ACU + U. In both comparisons, c-Fos expression in multiple brain regions contributed to the contrast, and the hypothalamus contributed the most strongly to this contrast. *CON* control, *ACU* acupuncture treatment, *ACU* + *U* U0126 administration followed by acupuncture treatment. Abbreviations for each brain region are shown in Table 1.

For identification of the brain regions that differ in c-Fos expression between control and acupuncture treatment or between acupuncture and peripheral U0126 administration followed by acupuncture, the contrast task PLS analysis was applied. Then, two pairs of CON vs. ACU and ACU vs. ACU + U were compared. The results showed strongly differentiated patterns in the CON vs. ACU (Fig. 4A) and ACU vs. ACU + U (Fig. 4B), and these two pairs were similar. These distinct patterns were associated with c-Fos activation in multiple regions of the cortex, cerebral nuclei, hippocampus, thalamus, hypothalamus, midbrain, and medulla, and mainly driven by c-Fos activation in the hypothalamus in both comparisons.

### Generation of functional connectivity after acupuncture

**Figure 5:**
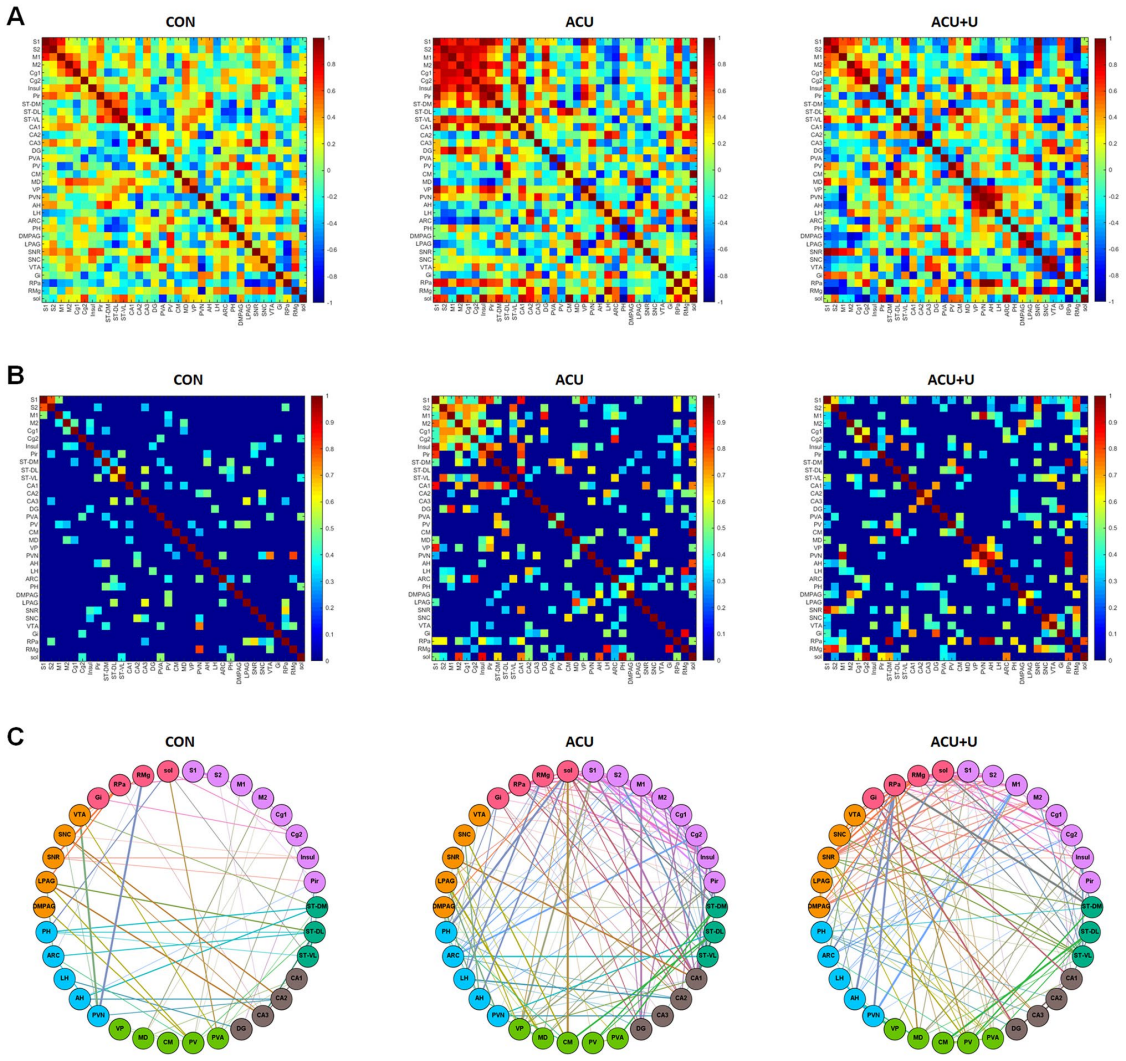
Matrices of inter-regional correlations and network construction for brain c-Fos expression within each group. (**A**) Colour-coded matrices showing inter-regional correlations for c-Fos activation between the 34 brain regions. (**B**) The threshold square of inter-regional correlations for c-Fos activation. Acupuncture produces a high correlation between the brain regions of the cortex, and these correlations were mostly altered by U0126 administration. Red colour indicates high correlation and blue indicates low-correlation (scale, right). (**C**) A circular layout grouped by major brain subdivision to show the connectivity between the brain regions. Nodes are connected by the edges of super-threshold inter-regional correlations. *CON* control; *ACU* acupuncture treatment, *ACU* + *U* U0126 administration followed by acupuncture treatment. Abbreviations for each brain region are shown in Table 1.

Next, we computed inter-regional pairwise correlations for c-Fos activation in the 34 brain regions. These analyses revealed that acupuncture produces a higher correlation between the brain regions, mainly in the cortex area, compared to the control. In some other brain regions of the hippocampus (CA1 and dentate gyrus; DG), cerebral nuclei (dorsal medial part of striatum; ST-DM and ventral lateral part of striatum; ST-VL), thalamus (ventral posterior thalamic nucleus; VP), hypothalamus (LH, ARC, and PH), and medulla (raphe pallidus nucleus; RPa, RMg, and Sol) also showed high correlation with other brain regions. Peripheral U0126 administration mostly diminished the increased correlation by acupuncture treatment. However, VP in the thalamus, PVN, and anterior hypothalamic area (AH) in the hypothalamus, RPa and RMg in the medulla, showed an increased correlation in the ACU + U group (Fig. 5A).

For better optimization of brain functional connectivity, we constructed brain networks by thresholding square of Pearson’s correlations in order to consider both positive and negative correlations, and the threshold was 0.288 which minimizes the number of edges when all brain networks have no isolated node (Fig. 5B). Then, the brain networks were visualized in a circular format (Fig. 5C). Brain regions (nodes) are connected by edges, indicating super-threshold inter-regional correlations. We found that acupuncture produced a high correlation between the brain regions compared to the control, and the ACU and ACU + U groups showed distinct patterns in brain functional connectivity. In the ACU group, the correlation between most cortical areas was increased. The cortical areas showed a high correlation with other brain regions, including the hypothalamus, thalamus, hippocampus, and cerebral nuclei. In particular, the extensive hypothalamic area (PH, ARC, AH, and LH), Sol of the medulla, CA1 of the hippocampus, and Cg2 of the cortex showed a strong connection with other brain regions. PH, LH, and AH regions of the hypothalamus showed strong connectivity with Sol and RMg of the medulla. In contrast, ARC showed strong connectivity with Cg2 of the cortex. Sol showed strong connectivity with not only the hypothalamus but also the central medial thalamic nucleus (CM) of the thalamus and CA1 of the hippocampus. Additionally, CA1 showed strong connectivity with the SNR of the midbrain, RPa, and Sol of the medulla and S1 of the cortex. The correlation patterns of the ACU + U group were significantly different from those of the ACU group. Most of the increased correlation between the brain regions by acupuncture was diminished by peripheral U0126 administration. However, a correlation between the midbrain and cortical area was observed, and the correlation between the thalamus and cerebral nuclei still existed in the ACU + U group. Additionally, RPa in medulla showed a strong correlation with AH and PVN in the hypothalamus and ST-DM in the cerebral nuclei in ACU + U group (Fig. 5C).

### Network clustering in force atlas format

**Figure 6:**
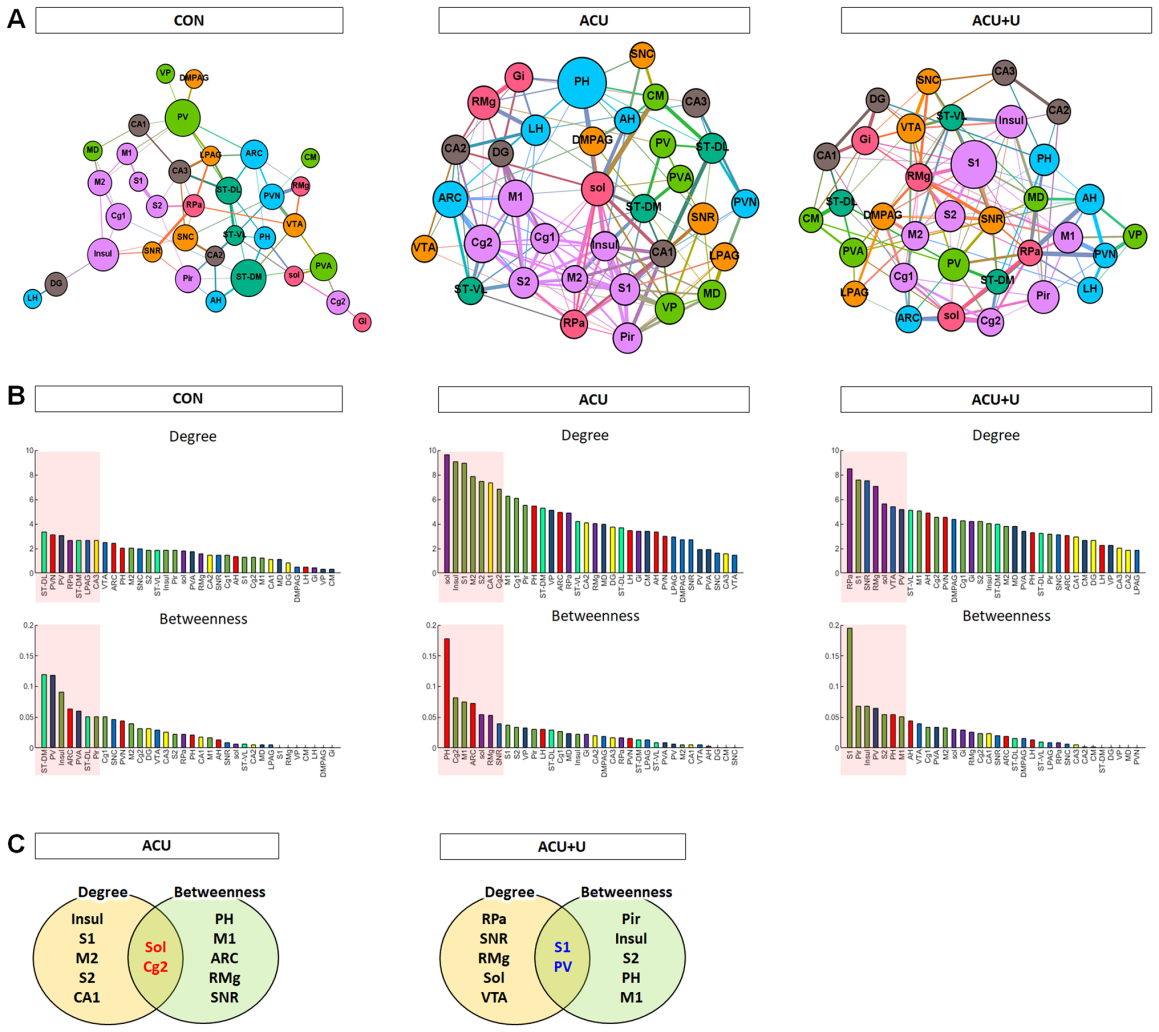
Network clustering and hub-identification. (**A**) A force atlas format to show the degree and betweenness centrality. The number of edges was represented as degree, and the number of shortest paths of all possible pairs of nodes represented betweenness centrality. (**B**) Brain regions were ranked in descending order for degree and betweenness centrality of the CON, ACU, and ACU + U groups. Brain regions ranked above the 80th percentile for degree and betweenness centrality were indicated by a red-coloured box. (**C**) Venn diagram shows the overlap between brain regions ranked above the 80th percentile for degree and betweenness centrality in the ACU and ACU + U groups. Two putative hub regions (Sol and Cg2 in the ACU group; S1 and PV in the ACU + U group) were identified as hub brain regions in each group. *CON* control, *ACU* acupuncture treatment, *ACU* + *U* U0126 administration followed by acupuncture treatment. Abbreviations for each brain region are shown in Table 1.

Then, we generated a force atlas format to show the degree and the betweenness centrality between the brain regions. In all results, the nodes were connected by edges, and the weighted number of edges was indicated as a degree. The betweenness centrality for each node is the weighted number of shortest paths that pass through the node from all shortest paths of all possible pairs of nodes. A high degree indicates that one brain region is connected to many other brain regions, and a high betweenness centrality indicates that one brain region is functionally correlated with other brain regions. After acupuncture, many edges between the regions were created. The number of edges was increased in Sol, Insul, S1, M2, secondary somatosensory cortex (S2), CA1, and Cg2 after acupuncture treatment. PH in the hypothalamus showed the highest betweenness centrality after acupuncture treatment, and Cg2, primary motor cortex (M1), ARC, Sol, RMg, and SNR also showed higher betweenness centrality after the acupuncture treatment. The ACU + U group showed an entirely different pattern from the ACU group. The number of edges was increased in RPa, S1, SNR, RMg, Sol, ventral tegmental area (VTA), and PV in ACU + U group, however, these values were lower than those in the acupuncture treated group. Various cortex areas showed high betweenness centrality in the ACU + U group. Among them, S1 showed the highest betweenness centrality (Fig. 6A,B).

These results indicate that the Sol is most connected to other brain regions, and the hypothalamic area containing PH and ARC played the most important role in the acupuncture-increased functional connectivity of the brain.

### Identification of hub brain regions

Identification of the putative hub regions that work as an important brain region mediating the brain neural mechanism of acupuncture, hub identification was performed. Brain regions were ranked in descending order for the degree and betweenness of all groups. In the ACU group, the regions of Sol, Insul, S1, M2, S2, CA1, and Cg2 were ranked above the 80th percentile for degree, and PH, Cg2, M1, ARC, Sol, RMg, and SNR were ranked above the 80th percentile for betweenness. Then, the overlap of ranked regions was selected. The two putative hub regions of Sol and Cg2 were selected as hub regions changed after acupuncture treatment. In the ACU + U group, RPa, S1, SNR, RMg, Sol, VTA, and PV were ranked above the 80th percentile for degree, and S1, Pir, Insul, PV, S2, PH, and M1 were ranked above the 80th percentile for betweenness. The two putative hub regions of S1 and PV were selected as hub regions that reacted with peripheral U0126 administration followed by acupuncture treatment (Fig. 6C).

## Discussion

Here, we found that peripheral ERK expression by acupuncture plays a triggering role in initiating acupuncture-induced peripheral proteomic changes. And we also found that the brain neural activation and its functional connectivity by acupuncture is regulated by peripheral ERK expression by acupuncture. To the best of our knowledge, this is the first study to explore the brain functional connectivity of acupuncture and to identify the peripheral molecular signaling initiating the central neural activity.

Since acupuncture works by inserting and/or manipulating a needle at a specific acupoint, the peripheral biomolecular changes after acupuncture may play an important role in initiating the therapeutic effect of acupuncture. Previously, several studies reported that peripheral biomolecules such as TRPV1^19,20^, adenosine A1 receptor^21^, or mast cell degranulation^23,24^ change after acupuncture stimulation. We previously identified that peripheral ERK signaling, a major molecule activated on the skin tissue after acupuncture treatment, plays a key role in mediating the analgesic effect of acupuncture^22^. In this study, peripheral U0126 administration blocked the expression of acupuncture-induced p-HSP27 activation, while the TRPV1 inhibitor and A1R inhibitor did not affect the acupuncture-induced peripheral ERK expression (Fig. 1D,E). Based on these results, we confirmed that peripheral ERK expression acts as an upstream regulator of peripheral biomolecular expression due to acupuncture treatment.

Analysis of brain functional connectivity is used as a useful method to identify the specific effects of acupuncture. Several studies have reported that acupuncture stimulation at different acupoints induces differential brain functional connectivity in functional MRI (fMRI)^25,26^. Recently, altering functional properties of brain networks using graph theory^27,28^ and hub configurations in some brain regions^29^ were investigated in advance. These studies have commonly used the degree and mean value to perform hub identification. Despite the similarities between structural and functional networks^30^, it is difficult to detect direct neurophysiological evidence among large-scale brain networks because of their variability in statistical measurements. In an effort to observe more direct brain neuronal responses, the expression of c-Fos activity as an immediate early gene has been utilized to quantify brain neural activity at a single-cell unit^18,31–33^. Previous studies have indicated that acupuncture induced c-Fos activation in some brain regions, including the ARC, ventrolateral periaqueductal grey (VLPAG), Sol, and RMg^34–37^. However, these studies only showed a partial indication to central nervous system changes, which limits the understanding of the broad spectrum of neural mechanisms regarding acupuncture. Therefore, it is necessary to observe whole-brain level analysis of neural activity and its functional connectivity after acupuncture treatment to improve our knowledge of the mechanisms of acupuncture.

In our results, the number of c-Fos-positive cells in S1, M2, Cg2, Insul, Pir, CA1, PV, LH, ARC, DMPAG, LPAG, and Sol were changed significantly after acupuncture treatment, and these changes were all inhibited by peripheral U0126 administration (Fig. 3). These results indicate that peripheral proteomic changes, especially ERK expression after acupuncture treatment, provoke brain neural changes. When we sorted the c-Fos changes by acupuncture and peripheral U0126 administration followed by acupuncture in order of change, ARC and LH were determined as the most affected brain areas by acupuncture and peripheral U0126 administration followed by acupuncture. Next, we performed salience analysis using the contrast task PLS method to identify the brain regions that are more affected by acupuncture than control or peripheral U0126 administration followed by acupuncture (Fig. 4). Analysis showed that CON vs. ACU and ACU vs. ACU + U showed strongly differentiated patterns in the contrast task PLS analysis, and the two patterns were quite similar. These similar patterns indicate that the brain areas altered by acupuncture, such as the cortex, cerebral nuclei, hippocampus, thalamus, hypothalamus, midbrain, medulla, and especially the hypothalamic area, coincide with the brain areas affected by peripheral U0126 administration. Among these brain areas, the hypothalamus showed the most differentiated pattern. These results are consistent with the previous results of this study, where the brain area most significantly altered by acupuncture and peripheral U0126 administration was the hypothalamus.

Next, we computed inter-regional pairwise correlations for c-Fos activation in the 34 brain regions and constructed brain networks in a circular format to explore the functional connectivity between each brain region altered by acupuncture (Fig. 5). We found that acupuncture produced a higher correlation between the brain regions. The correlation between most cortical areas increased, and the cortical areas had a high correlation with the hypothalamus, thalamus, hippocampus, and cerebral nuclei. Among them, PH, ARC, AH, and LH in the hypothalamus, Sol in the medulla, CA1 in the hippocampus, and Cg2 in the cortex showed a strong correlation with other brain regions. The ACU + U group showed different correlation patterns from that of the ACU group. Most of the acupuncture-increased correlation between the brain regions was diminished by peripheral U0126 administration, while some correlations between the midbrain and cortical area and the thalamus and cerebral nuclei were still observed. Unlike the ACU group, RPa in medulla showed strong correlation with AH and PVN in the hypothalamus and ST-DM in cerebral nuclei. These results suggest that the increased specific functional connectivity between the brain regions by acupuncture may be primarily involved in the clinical effects of acupuncture. Both our previous and current studies show that peripheral U0126 administration blocks the analgesic effects of acupuncture, suggesting that the peripheral biomolecular changes and the central neural activity and its functional connectivity are closely linked. Other brain regions that maintained an increase by peripheral ERK blockade might be associated with the mechanical stimulation of acupuncture rather than its therapeutic effects and is also thought to be related to the non-specific effects of acupuncture.

A force atlas format showed that acupuncture produced prominent functional brain connectivity compared to the control (Fig. 6A,B). The control group had fewer edges between the brain regions and showed a scale-free network. The ACU group had many edges unlike the control group, indicating that the connectivity between the brain regions increased after acupuncture. The extensive area of the cortex was highly connected between the regions, and they were closely connected with some key areas of ARC in the hypothalamus, Sol and RPa in the medulla, and CA1 in the hippocampus. The PH in the hypothalamus showed the highest betweenness centrality after acupuncture. These results are also consistent with our previous results of this study, with the hypothalamus being the most important region mediated by acupuncture. The ACU + U group showed lower values of degree and betweenness centrality than the ACU group, and showed distinct patterns compared to the ACU group in the force atlas format. In the ACU + U group, the high connectivity between cortical areas was diminished, and instead of the hypothalamic area, S1 of the cortex showed the highest betweenness centrality. Thus, these results suggest that acupuncture induces changes in brain functional connectivity patterns, and these changes are mediated by peripheral ERK activation. Additionally, the hypothalamus may be closely related to the therapeutic effect of acupuncture, and the S1 region maintained in the ACU + U group is considered to be related only to acupuncture stimulation. These stimuli-related brain regions might be associated with the non-specific effect of acupuncture rather than its therapeutic effects.

In our findings, the hypothalamic area has been derived as the major brain region that mediates the acupuncture effect. In brain neural activity analysis, hypothalamic area of ARC, and LH were the most affected brain regions by acupuncture and peripheral U0126 administration. When we analysed distributed patterns between the CON vs. ACU and ACU vs. ACU + U in contrast PLS analysis, the two pairs of patterns were quite similar. The hypothalamic area contributed most significantly to the distinct pattern changes in the brain region in both comparisons. Additionally, PH in the hypothalamus showed the strongest betweenness centrality after acupuncture. From these results, we concluded that the hypothalamus is not only the brain region where brain neuronal activities are most remarkably changed, but also the brain region that is most closely connected to other brain regions.

The hypothalamus, a crucial brain region that modulates the endocrine, autonomic, and central nervous system^38^, is located under the thalamus and anatomically linked to the anterior cingulate cortex, amygdala, hippocampus, and midbrain area. Based on these anatomical connections, the interconnected prefrontal-limbic network is strongly related to depressive disorders^39^, controlling food intake^40,41^, hypertension^42^, and pain^43,44^. Additionally, the hypothalamus is a representative brain region that is activated by acupuncture and is known to be closely associated with the various therapeutic effects of acupuncture, such as pain, chronic stress, and alcohol dependence^45–47^. The hypothalamic area is where closely related orexigenic neuropeptide and melanin-concentrating hormone (MCH) has been recently investigated in the role of acupuncture effects. We previously revealed that hypothalamic MCH mediated the neuroprotective action of acupuncture in a Parkinsonian mouse model^48^ and also promoted the analgesic effect in a persistent neuropathic mouse model^49^. Chen et al.^50^ also reported that median nerve stimulation, which is similar to PC6 acupuncture treatment, activated hypothalamic orexin neurons, and it mediates acupuncture analgesia through a cannabinoid 1 receptor-dependent cascade. Therefore, acupuncture-activated hypothalamic neuronal activation is closely related to the therapeutic effect of acupuncture in various neuronal diseases such as pain or neurodegenerative disease.

Finally, we derived the hub region, representing both high degree and high betweenness centrality. Recent brain network studies using graph theory have typically used degree and mean values to identify hub brain regions^27–29^. A degree indicates how many different brain regions are connected to a particular brain region. This can determine the extent of connections between brain regions, but it is difficult to determine how closely each brain region is connected. Since the betweenness centrality is a measure of how closely connected each brain region is^51^, we included not only degrees but also betweenness centrality for hub identification. Hub identification indicated that two regions of Sol and Cg2 have a major role in acupuncture-induced functional connectivity (Fig. 6C). Sol is an important primary nervous center for receiving somatosensory processes in the medulla and has extensive connections with many other structures in the CNS^35,37^. Sol is also the parasympathetic centre of the vagal pathway and transmits various sensory information from the viscera to the brain via afferent vagus fibers. Then, Sol integrates information from various brain areas and sends a signal to regulate the gastrointestinal (GI) tract via efferent vagal nerves^52,53^. Fang et al. demonstrated that electroacupuncture (EA) on ST36 and ST37 induced significant neuronal activity in Sol, which participates in different EA effects by acupoint and frequency selection^52^. Other previous studies also suggested that Sol contributed to the therapeutic effect of acupuncture on inflammation, regulating the GI tract, and relieving pain^54–57^. The cingulate cortex receives neuronal information from the thalamus and the neocortex, and then projects them to other brain areas of the prefrontal cortex, amygdala, hippocampus, hypothalamus, and midbrain^58,59^. These interconnected prefrontal-limbic networks are strongly involved in the formation and processing of emotion, learning, and memory, and these are related to acupuncture signaling transduction, controlling pain, and depression^38,39,60^. The cingulate cortex is known to be related to the mechanism of action of acupuncture in Parkinson’s disease, ischemic stroke, depressive disorders, and pain^46,48,61–65^.

**Figure 7:**
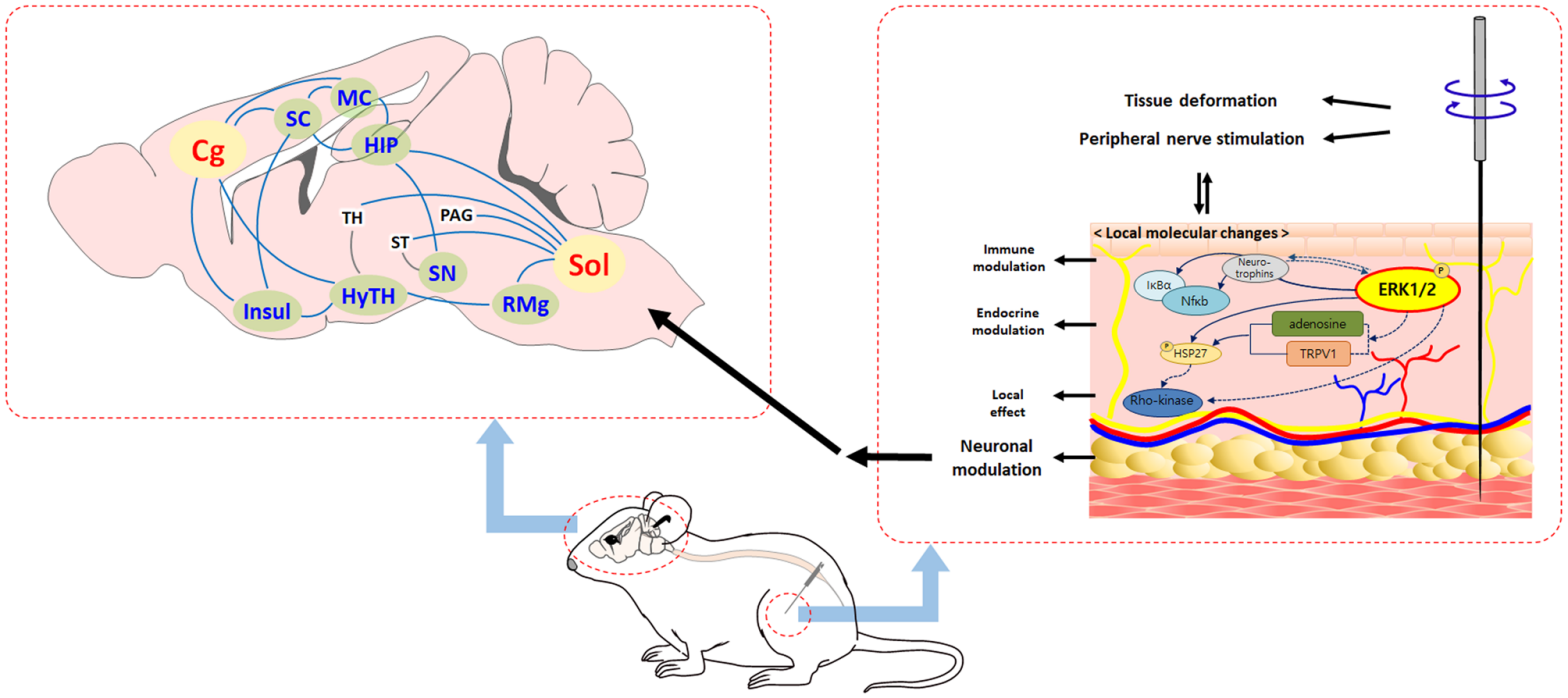
Schematic diagram of local molecular signaling and brain function after acupuncture treatment. The peripheral mechanism (right) and central mechanism (left) after acupuncture treatment are illustrated. Acupuncture-induced ERK1/2 activation acts as a triggering molecule that induces multiple signaling transduction around the acupoint. These local signals are received by the peripheral nerve ending and then transmitted to the afferent sensory nervous tract. The brain regions of Sol and Cg are mainly involved in acupuncture-mediated brain functional connectivity, and these brain regions are functionally connected with variable brain regions of SC, MC, HIP, Insul, HyTH, SN, and RMg. These central signals might activate the descending inhibitory pathway that provokes the acupuncture effect. The solid line is the mechanism of the findings in this study, and the dotted line is the potential prediction mechanism. *Cg* cingulate cortex, *Insul* insular cortex, *HIP* hippocampus, *HYTH* hypothalamus, *MC* motor cortex, *PAG* periaqueductal grey, *RMg* raphe magnus nucleus, *SC* somatosensory cortex, *SN* substantia nigra, *Sol* nucleus of solitary tract, *ST* striatum, *TH* thalamus.

Based on our results, it is thought that acupuncture can contribute to the treatment of numerous diseases by modulating major brain areas such as the hypothalamic area, Sol, and cingulate cortex. Brain regions that were not affected by peripheral U0126 administration were thought to be associated with the non-specific effect of acupuncture. From the results of this study, we suggest a hypothesis of acupuncture-induced peripheral and central mechanisms (Fig. 7). Acupuncture treatment produces peripheral biomolecular changes triggered by ERK activation, and peripheral signaling is received mainly by peripheral nerve endings, which are then transmitted to the afferent sensory nervous tract. Sol and RMg receive mechanical signals from the spinal cord and are functionally transmitted to the hippocampus, thalamus, PAG, ST, and hypothalamus. Then signals are sent to Cg, SC, MC, and Insul. These signals are then sent back to Sol and RMg and might activate the descending inhibitory pathway to provoke the acupuncture effect. The activation of c-Fos-positive cells, which are not affected by peripheral U0126 administration, might be due to the mechanical stimulus of needle insertion.

This study aims to investigate the discursive brain neuronal activity and brain functional connectivity after acupuncture and identify that these brain neural changes were exerted by peripheral molecular signaling. However, the study still has the limitation that no whole-brain areas have been observed and no more samples have been obtained for more rigorous functional connectivity analysis. In addition, there is a limitation that the control group for acupuncture stimulation, such as non-acupoint stimulation, was not used for brain functional connectivity analysis. For further study, it is necessary to include more brain regions to identify the most important brain region within the acupuncture-induced functional network. It is also necessary to investigate the functional network in the disease model with non-acupoint treated controls to determine the central therapeutic mechanism of acupuncture. Additionally, it is necessary to verify whether the brain areas derived from this study actually play an important role in mediating the therapeutic effect of acupuncture. These further studies could elicit a hypothesis of whole-brain functional connectivity related to the therapeutic effect of acupuncture.

In conclusion, acupuncture-induced peripheral ERK expression plays a triggering role in producing acupuncture-induced peripheral biomolecular signals. The neuronal activity and functional connectivity were noticeably changed after acupuncture, and these changes were altered by peripheral U0126 administration. The hypothalamic area was the most affected by acupuncture and peripheral U0126 administration. As a result of hub identification, Sol and Cg2 were derived as hub regions that reflect both degree and betweenness centrality after acupuncture. From these results, we have concluded that peripheral ERK expression after acupuncture plays a triggering role in initiating the acupuncture-induced peripheral biomolecular signaling pathway and also plays an important role in mediating brain neural activation and its functional connectivity.

## Materials and methods

### Experimental animals

Male C57BL/6 mice (8–9 weeks of age, weighing 20–25 g; Samtaco, Seoul, Korea) were used in all experiments. The mice were maintained under a 12 h:12 h light/dark schedule with free access to food and water. The mice were randomly divided, and three to four mice were placed in each cage (room temperature 20–25 °C and humidity of 40–60%) in a clean room. This study was carried out in accordance with the guidelines for the Care and Use of Laboratory Animals of the National Institutes of Health, and approved by the Kyung Hee University Animal Care Committee for animal welfare [KHUASP(SE)-13-053].

### Drugs and chemicals

U0126 (MEK/MAPK inhibitor, 8 μg/10 μl; Promega, Madison, WI, USA), CPZ (TRPV1 inhibitor, 0.4 μg/10 μl; Sigma, St. Louis, MO, USA), DPCPX (A1R inhibitor, 3 μg/10 μl; Tocris Bioscience, Bristol, BS110 QL, UK) were used to inhibit molecular activation in the skin layer. Each solution was injected into the GB34 acupoint 15 min before acupuncture treatment according to the previously described^66^. Briefly, mice were slightly anesthetized with ether, then each inhibitor was injected into the GB34 acupoint (i.d., 10 µL/each acupoint) using a 0.5 mL syringe (BD Biosciences, San Jose, CA, USA). The control mice were injected with an equal volume of vehicle.

### Acupuncture treatment

Acupuncture was performed at bilateral GB34 (Yangneungcheon) or non-acupoint on the hips for 10 min. GB34, located in the depression anterior and distal to the head of the fibula, is known to have an effect on motor dysfunction and pain (Fig. 1)^67–69^. The mice were immobilized in a clear acrylic holder and acupuncture needles (8 mm in length and 0.18 mm in diameter; Haenglim-seoweon Acuneedle Co., Gyeonggi-do, Republic of Korea) were inserted to a depth of 3 mm. Then, needles were turned at a rate of two spins per second for 10 s (one spin consists of 180° clockwise rotation and 180° counter-clockwise rotation) and removed 10 min later. The control mice were immobilized in a clear acrylic holder for 10 min to induce an equal amount of stress.

### Thermal nociceptive behavior

A hot plate test (Bioseb; Pinellas Park, FL, USA) was performed to evaluate thermal nociception^49^. The baseline measurement was conducted one day prior to the test measurements. For the acupuncture treatment, mice were immobilized in acryl holder, and acupuncture needles were inserted bilaterally to the GB34 acupoint for 10 min. Needles were turned bi-directionally for 30 s at every 2.5 min. U0126 (8 μg/10 μL) or vehicle (dimethyl sulfoxide; DMSO) were intradermally injected 15 min before acupuncture treatment. The negative control mice received acupuncture treatment on non-acupoints on the hip. Thermal hyperalgesia was measured 30 min after the acupuncture needles were removed.

### Skin preparation and western blotting

For protein analysis, skin samples around the GB34 acupoints were extracted 5, 10, 30, or 60 min after acupuncture treatment (n = 3 in each group). Briefly, the hairs of legs were removed one day before the experiment, and the mice were anesthetized with ether after acupuncture treatment. The bilateral skin tissues around the GB34 acupoint (5 mm in diameter, consisting of epidermis and dermis) were collected, and were homogenized in a lysis buffer (CyQUANT; Invitrogen, Eugene, OR, USA) containing protease inhibitor and phosphatase inhibitor cocktail tablets. After that, the samples were centrifuged for 15 min at 12,000 rpm, then the supernatants were collected. The protein concentration was calculated using a BCA assay. Western blot analysis was performed according to the previous study^22^. Briefly, 10 µg of total protein were separated using a 10% sodium dodecyl sulfate–polyacrylamide gel electrophoresis, then transferred to a PVDF membrane (Millipore, Billerica, MA, USA). The membranes were blocked with 5% non-fat dry milk in Tris buffered saline for 1 h, then incubated with the primary antibodies such as rabbit p-ERK, total ERK (t-ERK), p-HSP27, total HSP27 (t-HSP27), PRDX1 (Cell Signaling Technology, Beverly, MA, USA) and β-actin (Sigma, St. Louis, MO, USA) overnight at 4 °C. Then, membranes were incubated with secondary antibodies (horseradish peroxidase-conjugated goat anti-rabbit antibody or anti-mouse antibody; Pierce, Rockford, IL, USA) at room temperature. The protein expression was visualized using a chemiluminescence kit (Super Signal West Pico; Pierce, Rockford, IL, USA).

### Two-dimensional electrophoresis and peptide mass fingerprinting (PMF)

Skin tissues were homogenized in a lysis solution composed with 2 M Thiourea containing 4% (w/v) 3-[(3-cholamidopropy)dimethyammonio]-1-propanesulfonate (CHAPS), 7 M urea, 2% (v/v) pharmalyte and 1 mM benzamidine, and 1% (w/v) dithiothreitol (DTT). Proteins were extracted for 1 h with vortexing, centrifuged at 15,000*g* for 1 h, and soluble fraction was collected. The amount of protein was calculated using a Bradford method. IPG dry strips (4–10 NL IPG, 24 cm, Genomine, Korea) were equilibrated with 2 M thiourea containing 2% 3-[(3-cholamidopropy)dimethyammonio]-1-propanesulfonate (CHAPS), 7 M urea, 1% pharmalyte, and 1% dithiothreitol (DTT) for 12–16 h, and loaded with 200 ug of sample. Isoelectric focusing (IEF) was performed using EPS 3500 XL power supply and Multiphor II electrophoresis unit (Amersham Biosciences) according to the manufacturer’s instruction. Equilibrated strips were inserted onto SDS-PAGE gels (10–16%, 20 × 24 cm) and the SDS-PAGE was performed with a Hoefer DALT 2D system (Amersham Biosciences) according to the manufacturer’s instructions. 2D gels were run for 1700 Vh then stained with coomassie G250 as previously described^70^. Digitized images were analysed with PDQuest (version 7.0, BioRad) software according to the manufacturer’s protocols. Each spot was normalized by total spot intensity, then the protein expression level over two-fold compared with control sample was selected. Protein spots were excised, digested with trypsin (Promega, Madison, WI), and mixed with α-cyano-4-hydroxycinnamic acid in 50% acetonitrile / 0.1% TFA. Then they were subjected to MALDI-TOF analysis (Microflex LRF 20, Bruker Daltonics) for identification by peptide mass fingerprinting as previously described^71^. Spectra were collected from 300 shots per spectrum over m/z range 600–3000 and calibrated with a two-point internal calibration with trypsin auto-digestion peaks (m/z 842.5099, 2211.1046). The peak list was produced by Flex Analysis 3.0 (threshold: 500 for minimum resolution of monoisotopic mass; 5 for S/N), and the protein identification was performed by peptide mass fingerprinting with the search program MASCOT (http://www.matrixscience.com). The parameters were used for the database search as follows: a maximum of one missed cleavage, iodoacetamide (Cys) as a complete modification, trypsin as the cleaving enzyme, monoisotopic masses, oxidation (Met) as a partial modification, and a mass tolerance of ± 0.1 Da.

### Immunohistochemistry

Mice were deeply anesthetized 90 min after acupuncture, then perfused transcardially using phosphate buffered saline (PBS) followed by 4% paraformaldehyde (PFA). Brains were removed, post-fixed overnight, then cryoprotected in 30% sucrose for three days. Brains were cut into 40 μm using cryocut microtome (Leica Microsystems Inc., Tokyo, Japan) and prepared for free-floating sections. The brain sections were incubated in 1% hydrogen peroxide for 15 min, incubated with 5% normal goat serum in PBS for 1 h. Then the brain sections were incubated with primary antibody (rabbit c-Fos antibody; Santacruz Biotechnology, CA, USA) overnight at 4 °C, then incubated with secondary antibody (biotinylated anti-rabbit IgG; Vector Laboratories Inc., Burlingame, CA, USA) for 1 h. Then, they were incubated with ABC reagent (Vector Laboratories Inc., Burlingame, CA, USA) followed by 0.02% diaminobenzidine and 0.003% hydrogen peroxide in 1 M Tris-buffered saline (pH 7.5). After that, the sections were dehydrated in ethanol (95% and 100%), incubated in xylene, and mounted. Protein expression was pictured using microscope (BX51; Olympus, Tokyo, Japan).

The number of c-Fos positive cells was analysed in 34 brain regions of cortical, cerebral nuclei, hippocampal, thalamic, hypothalamic, midbrain, and medulla regions^72^. The c-Fos positive cells were quantified bilaterally for 2–3 times in each 2.5 mm^2^ tissue area using Image Pro Plus 6.0 software (Media Cybernetics, Inc., Silver Spring, MD, USA), then a mean value was computed. All procedures were performed blind to minimize the observer bias.

To find the brain regions most affected by acupuncture or local ERK inhibition, all controls were converted to 100%, and then the c-Fos increases in ACU groups were arranged in order. In the same way, all ACU groups were converted to 100%, and the c-Fos decreases in ACU + U groups were arranged in order. Then, the overlap between the brain regions ranked above the 80th percentile was selected.

### Contrast task PLS analysis (salience analysis)

The partial least squares (PLS) analysis, a multivariate statistical technique was used to identify optimal patterns of functional activity that differentiate experimental conditions^73^. Contrast task PLS was used to analyse the brain region activity to describe the relationship between central c-Fos activity and sets of contrasts which represent specific experimental conditions (CON, ACU, and ACU + U). There were three experimental groups in this study, but only two pairs of ACU vs. CON and ACU vs. ACU + U were compared. Brain measurement were stored in an $n \times m$ matrix denoted ${\text{X}}$ where the rows are observations and the columns are brain regions. Matrix ${\text{X}}$ is made up of two priori sub-matrices, with $n_{1} ,{ }n_{2}$ being the number of observations in two sub-matrices. The set of contrasts were stored in an $n \times l$ matrix ${\text{Y}}$. The contrasts are pairwise orthogonal and the sum of squares of a given contrast is equal to one. The matrix ${\text{Y}}$ is made up of the same two priori sub-matrices as ${\text{X}},$ with $l$ contrasts that code for different aspects of the experimental design. Because there are two experimental groups in each pair, the maximum number of orthonormal contrasts is one, i.e. $l = 1$. The relationships between ${\text{X}}$ and ${\text{Y}}$, denoted R, were stored in a cross-product matrix:

$${\text{R}} = {\text{Y}}^{{\text{T}}} {\text{X}}{.}$$

The singular value decomposition (SVD) of ${\text{R}}$ decomposes it into three matrices:

$${\text{R}} = {\text{U}}\Delta {\text{V}}^{{\text{T}}}$$

where ${\text{U}},{\text{ V}},{ }\;{\text{and }}\;\Delta$ are the normalized left singular vectors, the normalized right singular vectors, and diagonal matrix of the singular values, respectively. The singular vectors ${\text{U}}$ and ${\text{V}}$ are called saliences. The left singular vectors ${\text{U}}$ represent the experimental design that best characterize ${\text{R}}$, whereas the right singular vectors ${\text{V}}$ represent the brain measurement that best characterized R. In this paper, two saliences representing brain measurement, V of ‘ACU vs CON’ and ‘ACU vs ACU + U’, were compared. These saliences identified the brain regions showing the activation profile across tasks, indicating the maximally expressed brain areas in differences between pairs of experimental groups.

### Network generation

Within each of the three experimental groups (CON, ACU, and ACU + U), all possible pairwise correlations between the c-Fos expression in the 34 regions were determined by computing Pearson correlation coefficients. Each complete set of correlations was computed from each vector and were color-coded correlation matrices using MATLAB 2018a (version 9.4, http://www.mathworks.com/). Networks were constructed by thresholding square of correlations in order to consider both positive and negative correlations. In this procedure, an optimization algorithm was applied to minimize the number of edges when all networks had no isolated nodes. Then, the graph data were visualized using Gephi (version 0.9.2, https://gephi.org/).

### Functional connectivity analysis

Two measurements of network in this paper are usually used in brain connectivity analysis, and these are used to detect central brain regions in many previous studies.

The degree represents the number of nodes to which a focal node is connected. It measures the involvement of the node in the network. This is the most basic network measurement, and almost all other measurement are related to degree.

The betweenness centrality is the number of shortest paths that pass through that node from all shortest paths of all possible pairs of nodes^18,74,75,76^. The betweenness centrality of a node ‘$i$*’* is given as:

$$C_{i}^{B} = \frac{1}{N(N - 1)}\mathop \sum \limits_{s \ne i \ne t} \frac{{\rho_{st} (i)}}{{\rho_{st} }}$$

where ‘N’ is the number of all nodes, ‘$\rho_{st} (i)$’ is the total number of shortest paths between a source node ‘*s’* and a target node ‘*t’* that pass through node ‘*i’*. ‘$\rho_{st}$’ is the total number of all shortest paths between the node ‘*s’* and the node ‘*t’*^77^. All betweenness centralities were normalized.

The degrees and the betweenness centralities of every nodes in each network were measured. To show the connectivity between the brain regions effectively, a circular layout grouped by major brain subdivision was used^78^. And a force atlas format was used to show the degree and the betweenness centrality. Then, we organised degrees and betweenness centralities of each network to distribution tables for hub identification.

### Hub identification

Hub regions play important roles in the function of a network. Some previous researchers used the intersection of degree and betweenness centrality to identify hub regions in electrophysiology studies or large-scale brain imaging^77^. In this study, we selected brain regions that are represented as above 80% of both degree and betweenness centrality.

### Statistical analysis

All the data were expressed as the mean ± standard error of the mean (SEM), and analysed using GraphPad Prism 5 software (GraphPad Software Inc., San Diego, CA, USA) for the statistical analysis. For the behaviour test, western blotting, and immunostaining, data were analysed using one-way ANOVA with the Newman-Keuls post-hoc test. The differences between the data were considered statistically significant at *P* < 0.05.

## Supplementary Information

Supplementary Information.

## Data Availability

The datasets used in this manuscript and all analysed data from this study are available from the corresponding author upon reasonable request.

## References

[1] Woollam CH, Jackson AO (1998). Acupuncture in the management of chronic pain. Anaesthesia.

[2] Mavrommatis CI, Argyra E, Vadalouka A, Vasilakos DG (2012). Acupuncture as an adjunctive therapy to pharmacological treatment in patients with chronic pain due to osteoarthritis of the knee: A 3-armed, randomized, placebo-controlled trial. Pain.

[3] Joh TH, Park HJ, Kim SN, Lee H (2010). Recent development of acupuncture on Parkinson's disease. Neurol. Res..

[4] Zeng BY, Salvage S, Jenner P (2013). Effect and mechanism of acupuncture on Alzheimer's disease. Int. Rev. Neurobiol..

[5] Lam YC (2008). Efficacy and safety of acupuncture for idiopathic Parkinson's disease: A systematic review. J. Altern. Complement Med..

[6] Kim HY (2009). Electroacupuncture suppresses capsaicin-induced secondary hyperalgesia through an endogenous spinal opioid mechanism. Pain.

[7] Gim GT (2011). Electroacupuncture attenuates mechanical and warm allodynia through suppression of spinal glial activation in a rat model of neuropathic pain. Brain Res. Bull..

[8] Deng S (2015). Is acupuncture no more than a placebo? Extensive discussion required about possible bias. Exp. Ther. Med..

[9] Kong J (2009). Expectancy and treatment interactions: A dissociation between acupuncture analgesia and expectancy evoked placebo analgesia. Neuroimage.

[10] Harris RE (2009). Traditional Chinese acupuncture and placebo (sham) acupuncture are differentiated by their effects on mu-opioid receptors (MORs). Neuroimage.

[11] Kong J (2009). An fMRI study on the interaction and dissociation between expectation of pain relief and acupuncture treatment. Neuroimage.

[12] Yu SW (2019). Acupuncture effect and mechanism for treating pain in patients with Parkinson's disease. Front. Neurol..

[13] Tu Y (2019). Multivariate resting-state functional connectivity predicts responses to real and sham acupuncture treatment in chronic low back pain. NeuroImage Clin..

[14] Cai RL, Shen GM, Wang H, Guan YY (2018). Brain functional connectivity network studies of acupuncture: A systematic review on resting-state fMRI. J. Integr. Med..

[15] Sporns O (2013). Structure and function of complex brain networks. Dialogues Clin. Neurosci..

[16] Rubinov M, Bullmore E (2013). Schizophrenia and abnormal brain network hubs. Dialogues Clin. Neurosci..

[17] Aggleton JP, Brown MW, Albasser MM (2012). Contrasting brain activity patterns for item recognition memory and associative recognition memory: Insights from immediate-early gene functional imaging. Neuropsychologia.

[18] Wheeler AL (2013). Identification of a functional connectome for long-term fear memory in mice. PLoS. Comput. Biol.

[19] Abraham TS, Chen ML, Ma SX (2011). TRPV1 expression in acupuncture points: Response to electroacupuncture stimulation. J. Chem. Neuroanat..

[20] Wu SY, Chen WH, Hsieh CL, Lin YW (2014). Abundant expression and functional participation of TRPV1 at Zusanli acupoint (ST36) in mice: Mechanosensitive TRPV1 as an "acupuncture-responding channel". BMC Complement. Altern. Med..

[21] Goldman N (2010). Adenosine A1 receptors mediate local anti-nociceptive effects of acupuncture. Nat. Neurosci..

[22] Park JY (2014). From peripheral to central: The role of ERK signaling pathway in acupuncture analgesia. J. Pain.

[23] Ding N (2018). Mast cells are important regulator of acupoint sensitization via the secretion of tryptase, 5-hydroxytryptamine, and histamine. PLoS ONE.

[24] Wang L (2013). ATP release from mast cells by physical stimulation: A putative early step in activation of acupuncture points. Evid. Based Complement. Altern. Med..

[25] Qin W (2011). The temporal-spatial encoding of acupuncture effects in the brain. Mol. Pain.

[26] Zheng Y (2016). Imaging of brain function based on the analysis of functional connectivity—Imaging analysis of brain function by Fmri after acupuncture at Lr3 in healthy individuals. Afr. J. Tradit. Complement. Altern. Med. AJTCAM.

[27] Liu B (2012). Altered small-world efficiency of brain functional networks in acupuncture at ST36: A functional MRI study. PLoS ONE.

[28] Zhang Y (2013). Long-duration transcutaneous electric acupoint stimulation alters small-world brain functional networks. Magn. Reson. Imaging.

[29] You Y (2013). Altered hub configurations within default mode network following acupuncture at ST36: A multimodal investigation combining fMRI and MEG. PLoS ONE.

[30] Caeyenberghs K, Leemans A, Leunissen I, Michiels K, Swinnen SP (2013). Topological correlations of structural and functional networks in patients with traumatic brain injury. Front. Hum. Neurosci..

[31] Ferraguti F (2019). An appraisal of the influence of the metabotropic glutamate 5 (mGlu5) receptor on sociability and anxiety. Front. Mol. Neurosci..

[32] Tanimizu T (2017). Functional connectivity of multiple brain regions required for the consolidation of social recognition memory. J. Neurosci..

[33] Tanimizu T, Kono K, Kida S (2018). Brain networks activated to form object recognition memory. Brain Res. Bull..

[34] Guo ZL, Longhurst JC (2010). Activation of reciprocal pathways between arcuate nucleus and ventrolateral periaqueductal gray during electroacupuncture: Involvement of VGLUT3. Brain Res..

[35] Chen CY (2013). The possible neuronal mechanism of acupuncture: Morphological evidence of the neuronal connection between groin A-Shi point and uterus. Evid. Based Complement. Altern. Med. eCAM.

[36] Kim SK (2010). Electroacupuncture induces Fos expression in the nucleus tractus solitarius via cholecystokinin A receptor signaling in rats. Neurol. Res..

[37] He JF (2013). Neuron discharge and c-Fos expression in the nucleus of the solitary tract following electroacupuncture at acupoints of the Yangming Stomach Meridian of Foot. J. Acupunct. Meridian Stud..

[38] Bao A, Meynen G, Swaab D (2008). The stress system in depression and neurodegeneration: Focus on the human hypothalamus. Brain Res. Rev..

[39] Bennett M (2011). The prefrontal–limbic network in depression: Modulation by hypothalamus, basal ganglia and midbrain. Prog. Neurobiol..

[40] Fukushima A (2015). Sex differences in feeding behavior in rats: The relationship with neuronal activation in the hypothalamus. Front. Neurosci..

[41] Duca FA, Yue JT (2014). Fatty acid sensing in the gut and the hypothalamus: In vivo and in vitro perspectives. Mol. Cell Endocrinol..

[42] Chen H (2013). Hypothalamus-related resting brain network underlying short-term acupuncture treatment in primary hypertension. Evid. Based Complement. Altern. Med..

[43] Coppola G (2019). Increased neural connectivity between the hypothalamus and cortical resting-state functional networks in chronic migraine. J. Neurol..

[44] Lerebours F (2019). Functional connectivity of hypothalamus in chronic migraine with medication overuse. Cephalalgia.

[45] Chang, S. *et al.* Acupuncture attenuates alcohol dependence through activation of endorphinergic input to the nucleus accumbens from the arcuate nucleus. *Sci. Adv.* **5**, 1342. 10.1126/sciadv.aax1342 (2019). 10.1126/sciadv.aax1342 PMC6726441 31517050

[46] Lee MJ (2019). Effects of acupuncture on chronic stress-induced depression-like behavior and its central neural mechanism. Front. Psychol..

[47] Yen, C. M., Wu, T. C., Hsieh, C. L., Huang, Y. W. & Lin, Y. W. Distal electroacupuncture at the LI4 acupoint reduces CFA-induced inflammatory pain via the brain TRPV1 signaling pathway. *Int. J. Mol. Sci*. 10.3390/ijms20184471 (2019). 10.3390/ijms20184471 PMC6769885 31510092

[48] Park JY (2016). Novel neuroprotective effects of melanin-concentrating hormone in Parkinson's disease. Mol. Neurobiol..

[49] Jang JH (2018). Novel analgesic effects of melanin-concentrating hormone on persistent neuropathic and inflammatory pain in mice. Sci. Rep..

[50] Chen YH (2018). Median nerve stimulation induces analgesia via orexin-initiated endocannabinoid disinhibition in the periaqueductal gray. Proc. Natl. Acad. Sci. U.S.A..

[51] Cheng H (2015). Nodal centrality of functional network in the differentiation of schizophrenia. Schizophr. Res..

[52] Fang JF, Du JY, Shao XM, Fang JQ, Liu Z (2017). Effect of electroacupuncture on the NTS is modulated primarily by acupuncture point selection and stimulation frequency in normal rats. BMC Complement. Altern. Med..

[53] Cui Y (2016). Electroacupuncture alleviates cisplatin-induced nausea in rats. Acupunct. Med.

[54] Lim HD, Kim MH, Lee CY, Namgung U (2016). Anti-inflammatory effects of acupuncture stimulation via the vagus nerve. PLoS ONE.

[55] Guo ZL, Malik S (2019). Acupuncture activates a direct pathway from the nucleus tractus solitarii to the rostral ventrolateral medulla. Brain Res..

[56] Zhang, F. *et al.* Neurobiological mechanism of acupuncture for relieving visceral pain of gastrointestinal origin. *Gastroenterol. Res. Pract.* **2017** (2017). 10.1155/2017/5687496 PMC5294365 28243252

[57] Lu MJ, Yu Z, He Y, Yin Y, Xu B (2019). Electroacupuncture at ST36 modulates gastric motility via vagovagal and sympathetic reflexes in rats. World J. Gastroenterol..

[58] Jacobson, S., Marcus, E. M. & Pugsley, S. *Neuroanatomy for the Neuroscientist* 477–529 (Springer, Berlin, 2018).

[59] Scheel, N., Wulff, P. & de Mooij‐van Malsen, J. G. Afferent connections of the thalamic nucleus reuniens in the mouse. *J. Comp. Neurol.* (2019). 10.1002/cne.24811 31721201

[60] Hayden BY, Platt ML (2010). Neurons in anterior cingulate cortex multiplex information about reward and action. J. Neurosci..

[61] Park JY (2017). Acupuncture modulates brain neural activity in patients: A systematic review and meta-analysis. Orient. Pharm. Exp. Med.

[62] Huang Y (2011). Needling at the waiguan (SJ5) in healthy limbs deactivated functional brain areas in ischemic stroke patients: A functional magnetic resonance imaging study. Neural Regen. Res..

[63] Chae Y (2009). Parsing brain activity associated with acupuncture treatment in Parkinson's diseases. Mov. Disord..

[64] Shao XM (2015). Strong manual acupuncture stimulation of “Huantiao” (GB 30) reduces pain-induced anxiety and p-ERK in the anterior cingulate cortex in a rat model of neuropathic pain. Evid. Based Complement. Altern. Med..

[65] Lee IS, Cheon S, Park JY (2019). Central and peripheral mechanism of acupuncture analgesia on visceral pain: A systematic review. Evid. Based Complement. Altern. Med..

[66] Dai Y (2002). Phosphorylation of extracellular signal-regulated kinase in primary afferent neurons by noxious stimuli and its involvement in peripheral sensitization. J. Neurosci..

[67] Mi WL (2011). Involvement of spinal neurotrophin-3 in electroacupuncture analgesia and inhibition of spinal glial activation in rat model of monoarthritis. J. Pain.

[68] Sun S (2008). Evidence for suppression of electroacupuncture on spinal glial activation and behavioral hypersensitivity in a rat model of monoarthritis. Brain Res. Bull..

[69] WHO, R. O. f. t. W. P. *WHO Standard Acupuncture Point Locations in the Western Pacific Region*. Vol. 1 (World Health Organization, Western Pacific Region, 2008).

[70] Anderson NL, Esquer-Blasco R, Hofmann JP, Anderson NG (1991). A two-dimensional gel database of rat liver proteins useful in gene regulation and drug effects studies. Electrophoresis.

[71] Fernandez J, Gharahdaghi F, Mische SM (1998). Routine identification of proteins from sodium dodecyl sulfate-polyacrylamide gel electrophoresis (SDS-PAGE) gels or polyvinyl difluoride membranes using matrix assisted laser desorption/ionization-time of flight-mass spectrometry (MALDI-TOF-MS). Electrophoresis.

[72] Keith B.J. Franklin, G. P. *The Mouse Brain in Stereotaxic Coordinates* (Academic Press in an imprint of Elsevier, 2008).

[73] McIntosh AR (1999). Mapping cognition to the brain through neural interactions. Memory.

[74] Rubinov M, Sporns O (2009). Complex network measures of brain connectivity: Uses and interpretations. Neuroimage.

[75] Goh KI, Oh E, Kahng B, Kim D (2003). Betweenness centrality correlation in social networks. Phys. Rev. E Stat. Nonlin. Soft Matter Phys..

[76] Lee SH (2013). Network analysis of acupuncture points used in the treatment of low back pain. Evid. Based Complement. Altern. Med..

[77] Sporns O, Honey CJ, Kotter R (2007). Identification and classification of hubs in brain networks. PLoS ONE.

[78] Irimia A, Chambers MC, Torgerson CM, Horn JD (2012). Circular representation of human cortical networks for subject and population-level connectomic visualization. Neuroimage.

