# Supplementary material for: Peripheral ERK modulates acupuncture-induced brain neural activity and its functional connectivity
Source: Sci Rep. 2021 Mar 4;11:5128. doi: 10.1038/s41598-021-84273-y (PMC7933175; doi:10.1038/s41598-021-84273-y)
Supplement: Supplementary file 1 — Supplementary Information. [file 41598_2021_84273_MOESM1_ESM.pdf]

Supplementary material

Peripheral ERK modulates acupuncture-induced brain neural activity and its functional connectivity

Ji-Yeun Park,[1] Seong-Jin Cho,[2] Soon-Ho Lee,[3] Yeonhee Ryu,[2] Jae-Hwan Jang,[3] Seung-Nam Kim[4] and Hi-Joon Park[3,*]

[1]College of Korean Medicine, Daejeon University, 62 Daehak-ro, Dong-gu, Daejeon 34520, Republic of Korea

[2]Clinical Medicine Division, Korea Institute of Oriental Medicine, 1672 Yuseong-daero, Yuseong-gu, Daejeon 34054, Republic of Korea

[3]Acupuncture and Meridian Science Research Center, Kyung Hee University, 26 Kyungheedae-ro, Dongdaemoon-gu, Seoul 02447, Republic of Korea

[4]College of Korean Medicine, Dongguk University, 32 Dongguk-Ro, Goyang 10326, Republic of Korea

*Corresponding author: Hi-Joon Park

Acupuncture and Meridian Science Research Center, Kyung Hee University, 26 Kyungheedae-ro, Dongdaemoon-gu, Seoul 02447, Republic of Korea.

E-mail: acufind@khu.ac.kr

Tel: +82-2-961-0371

Fax: +82-2-961-0916

Supplementary table 1. Acupuncture-induced protein candidates in two-dimensional electrophoresis analysis

Number of Standard spot	Molecular weight	Isoelectric point	Top scored protein	High scored protein
302	83.5	4.3	keratin, type II cytoskeletal 1b	
401	102.3	4.3	unnamed protein product	serum albumin precursor
1002	31.3	5.2	apolipoprotein A-I preproprotein	
1103	57.9	5.2	put. beta-actin	
1104	58.2	5.2	put. beta-actin	
1507	97.5	5.5	unnamed protein product	serum albumin precursor
4005	32.5	7.1	heat shock protein beta-1	
7010	16.2	8.0	unnamed protein product	hemoglobin beta
7501	109.1	8.0	Unknown	serotransferrin precursor, transferrin
8401	95.3	8.7	transketolase	
9005	30.9	9.8	unnamed protein product	peroxiredoxin-1

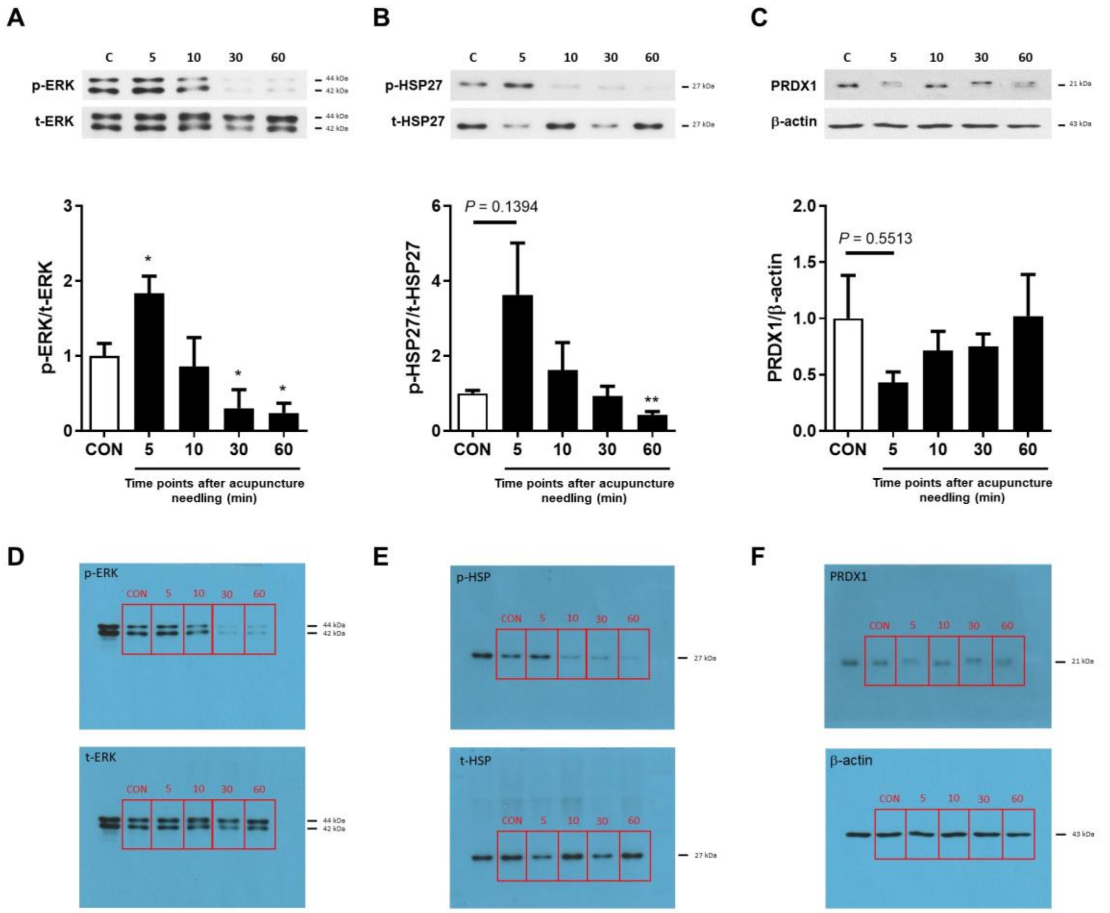

Supplementary figure 1. Confirmation of acupuncture-induced protein candidates identified by two-dimensional electrophoresis analysis

The activation of p-ERK and p-HSP27 was increased at 5 min after acupuncture (A, B) and the activation of PRDX1 was decreased at 5 min after acupuncture (C). (D-F) Blots of the cropped image of (A-C). The cropped areas are labelled with red-boxes. Each blots were cropped from the same gel. $^{*}P < 0.05$ compared to the CON group. One-way ANOVA was followed by the Newman-Keuls post-hoc test. Data are expressed as the mean ± SEM (each n = 3).

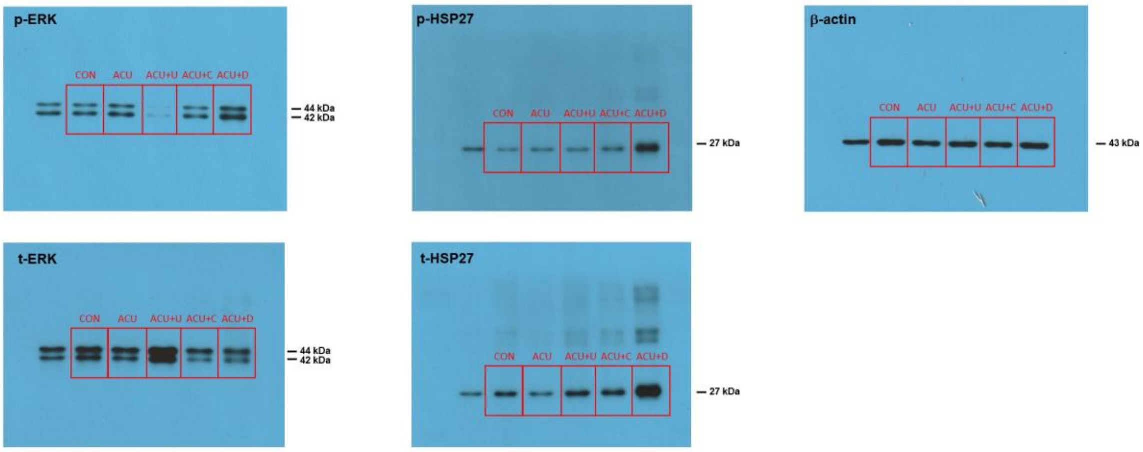

Supplementary figure 2. Blots of the cropped image shown in Figure 1D-E.

The cropped areas are labelled with red-boxes. Each blots were cropped from the same gel.
